# A laminin-based therapy for dogs with chronic spinal cord injury: promising results of a longitudinal trial

**DOI:** 10.3389/fvets.2025.1592687

**Published:** 2025-08-13

**Authors:** Carolina de Miranda Chize, Diego Gonzales Vivas, Karla Menezes, Max Nascimento Freire, Rodrigo Ferreira Pinto Jiddu, Aurélio Vicente Graça-Souza, Eliel de Souza-Leite, Paulo Roberto Louzada, Tatiana Coelho-Sampaio

**Affiliations:** ^1^Institute of Biomedical Sciences, Federal University of Rio de Janeiro, Rio de Janeiro, Brazil; ^2^Pathology Program, Federal University of Rio de Janeiro, Rio de Janeiro, Brazil; ^3^University Estacio de Sá, Rio de Janeiro, Brazil; ^4^Institute of Medical Biochemistry, Federal University of Rio de Janeiro, Rio de Janeiro, Brazil

**Keywords:** spinal cord injury, chronic, laminin, polylaminin, chondroitinase ABC, GDNF

## Abstract

**Introduction:**

Polylaminin, an improved form of the natural protein laminin, has been shown to promote axonal regeneration and functional recovery in animal models of acute spinal cord injury (SCI), and is safe and potentially beneficial in humans when administered within the first days after traumatic SCI. This study aimed to evaluate the effect of polylaminin in dogs with chronic SCI.

**Methods:**

We conducted a prospective, longitudinal study in six paraplegic dogs with severe chronic thoracolumbar SCI (T3-L3) caused by trauma (*n* = 2) or disc degeneration (*n* = 4). The study assessed whether gait scores, measured during an extended screening period (at least 4 months), would improve during the follow-up (6 months). Polylaminin was delivered intraspinally at a dose of 1 μg/kg, in combination with either glial-derived neurotrophic factor (GDNF; Group 1; *n* = 3) or chondroitinase ABC (Group 2; *n* = 3). Safety was assessed through neurological examinations, blood tests and monitoring of medical complications. Gait analysis was carried out using the Texas Spinal Cord Injury Scale (TSCIS) and the Open Field Scale (OFS), while a linear mixed model was used for statistical analysis. During the screening period, dogs received physiotherapy twice per week and had their gait scored periodically. The first six dogs whose scores had remained stable across three evaluations were enrolled. After owners provided informed consent, dogs were randomly allocated to either treatment group.

**Results:**

No neurological deterioration, serious clinical events or notable deviations in blood tests were observed. The TSCIS average baseline score increased from 2.2 to 3.2 (95% CI: 0.77–1.2; *p* < 0.001), while the OFS score increased from 1.5 to 3.1 (95% CI: 1.3–1.9; *p* < 0.001).

**Discussion:**

Although the present study could not discriminate between the benefits of the two treatments, our findings suggest that polylaminin, in combination with GDNF or chondroitinase ABC, is a safe and potentially effective treatment, which underscores the relevance of further studies to establish a new approach to improving gait function in dogs with chronic SCI.

## 1 Introduction

Dogs are common victims of naturally occurring SCI, not only due to external causes such as run-over injuries, but also due to the susceptibility of some breeds to intervertebral disc degeneration (IVDD), which compresses the spine, impairing normal ambulation (1, 2). As in humans, there are no treatments capable of inducing axonal regeneration after SCI in dogs, which justifies the search for new strategies to reverse the loss of ambulatory function in these animals.

Laminin is a natural extracellular matrix protein ubiquitously distributed in the animal body. It is the major constituent of basement membranes, a sheet-like type of matrix, involved in cell support and tissue compartmentalization (3). Under physiological conditions, laminin exists in a polymeric form, but such arrangement is lacking when the protein is extracted from biological tissues or when it is expressed in heterologous systems (4). Many years ago, our laboratory devised a protocol to restore the natural polymeric organization of laminin and named it polylaminin (polyLM) (5, 6). As the spontaneous regeneration of lesioned axons in the peripheral nervous system (PNS) occurs inside laminin-enriched tubular structures produced by Schwann cells (7), which are exclusive to PNS, we set out to investigate whether the provision of exogenous laminin in the polymeric form, polyLM, could induce axonal regeneration in the central nervous system (CNS) (8, 9). After demonstrating the enhanced axon growing properties of polyLM for CNS neurons *in vitro*, we used a rat model of acute spinal cord injury (SCI) to investigate whether an intraparenchymal injection of polyLM could also induce axonal regeneration *in vivo* (10). Since the observed effects of polyLM treatment encompassed the recovery of open field locomotion, we then conducted a pilot human trial to investigate the safety and possible benefits of polyLM treatment delivered during the acute phase of SCI. In that study, patients diagnosed with complete SCI received an intraparenchymal injection of polyLM within up to 6 days after trauma and were followed for 12 months. The treatment was considered safe and potentially beneficial because 75% of treated patients recovered some degree of motor function, while the rate of spontaneous motor recovery in cases of complete SCI does not typically exceed 15% (11).

The next step toward developing polylaminin as a treatment for SCI was to investigate its potential benefits in the chronic phase. To that end, we had to address two important challenges, namely the choice of an appropriate experimental model of chronic SCI, as discussed below, and the need to combine polyLM with an adjuvant treatment capable of overcoming the inhibitory effects of scarring,. The injury leads to the formation of a glial scar that extends beyond the margins of the original lesion and to a fluid-filled cavity surrounded by fibrotic tissue rich in inhibitory molecules, which impede axonal regeneration (12). To address these issues, we sought to combine polylaminin either with chondroitinase ABC (ChABC) to enzymatically remove the non-permissive scar component, chondroitin sulfate (13–15), or with glial cell–derived neurotrophic factor (GDNF), which has recently been identified as a potent chemoattractive factor for regenerating axons in experimental models of SCI (16, 17).

Given that the induction of long-term, stable functional deficits in rodent models often requires extremely severe spinal cord lesions—conditions typically associated with increased mortality rates and ethical concerns (18)—we chose to conduct a clinical trial in dogs, who may naturally develop permanent neurological sequelae following SCI. However, the likelihood of spontaneous ambulatory recovery in dogs with acute SCI is substantially higher than that observed in humans. For instance, a large retrospective study conducted in Japan involving 831 dogs with thoracolumbar intervertebral disc disease (IVDD) of varying severities found that 97.7% of animals with preserved deep pain perception (DPP), and 52.1% of those without DPP, regained ambulatory function within 2 months post-injury (19). Similar findings were previously reported by Olby et al. (20), who observed a 69% recovery rate in dogs with IVDD lacking DPP. The absence of DPP has been widely accepted as an indicator of complete lesions and is therefore routinely used to inform prognosis during the acute phase of SCI (21).

To evaluate the potential effects of polylaminin in the chronic phase of SCI, we specifically targeted the subset of animals that failed to recover spontaneous ambulation. Our inclusion criteria required the absence of functional improvement for a minimum of 3 months post-injury, followed by an additional four-month period of physical rehabilitation during which no gains in motor function were observed. This extended screening period ensured that only dogs with stable, non-recovering deficits were enrolled in the study, thereby minimizing the confounding influence of delayed spontaneous recovery. Moreover, it allowed the establishment of an accurate, individualized baseline of motor function for each animal prior to treatment, enabling within-subject comparisons of motor scores over time. This approach represents an optimal control design for a longitudinal clinical study, in which each subject serves as its own control. Additionally, it contributes to reducing the total number of animals required and allows the inclusion of a broader range of SCI presentations, thereby enhancing the external validity of the findings. Given the heterogeneous nature of the sample and the repeated measures across time, statistical significance was assessed using a robust analytical approach—namely, a linear mixed-effects model—which accounts for both fixed and random effects in the dataset.

The data presented here indicate that the administration of polyLM in combination with either ChABC or GDNF was safe and associated with a statistically significant improvement in gait performance in non-ambulatory dogs. Although the study design does not allow for definitive conclusions regarding the specific effect of polyLM—due to the absence of blinding and the inability to disentangle the individual contributions of polyLM and the adjuvant treatments—it provides evidence that motor deficits associated with SCI can be addressed even in the chronic phase. These findings offer valuable insights to inform future trials aimed at developing effective therapies for paralysis in both dogs and humans.

## 2 Materials and methods

### 2.1 Organization and design of the study

This was a prospective, longitudinal veterinary clinical trial designed to assess safety and a preliminary indication of efficacy of two combination treatments in dogs with chronic SCI. The protocol of the study was revised and approved by the Ethics Committee for Animal Use of the Health Sciences Center of the Federal University of Rio de Janeiro (CEUA-CCS/UFRJ), receiving the registration code 132/22.

Due to its exploratory nature, the study did not have a preestablished hypothesis and we defined a convenience sample of 6 participants. The overall structure of the trial comprised a long screening period, along which conventional therapies, covering orthopedic surgery (spinal decompression and/or vertebral stabilization) and physical therapy, were delivered to the patient, aiming at establishing the baseline performance of each individual dog before the treatment. The rationale of this design was to exclude the contribution of any concurrent treatment affecting gait, isolating the eventual gains produced by the experimental therapy.

Before initiation of the screening physiotherapy dogs received blood tests, cardiovascular evaluation and computed tomographic myelography (CTM). When feasible (when the animals had no MRI-incompatible materials present in the vertebral column), dogs were additionally submitted to magnetic resonance imaging (MRI) to precisely localize the neurological lesions. The animals that had not been submitted to previous conventional orthopedic surgery to decompress the spine and/or to stabilize the column received surgery before starting on the physical therapy program of the screening.

The physiotherapy sessions were carried out twice a week and included therapeutic exercises such as stretching, joint mobilization, neurosensory stimulation, neural mobilization and isometric exercises, dry needling, laser therapy and walking on conventional and/or underwater treadmill depending on each animal’s capacity. The duration of the screening depended on the evolution of each dog, accounting for the possibility that each dog could improve during the screening physiotherapy or not present any improvement. The screening was terminated when the gait scores were stabilized for 3 consecutive evaluations. The definition of stabilization was considered as obtaining either 3 equal scores or 3 scores varying less than 1 point (inclusive) without a trend for progression or regression. Animals that did not reach stabilization within a maximum of 5 months were not included in the study.

The signature of the informed consent form by the owners marked the transition between the screening and the clinical trial proper, which started by the administration of the experimental treatment. The present study tested two alternative combinatory treatments, namely polyLM + GDNF and ChABC + polyLM. The first three dogs to reach stabilization during the screening were allocated to group 1, randomly assigned to receive polyLM + GDNF and the next three were allocated to group 2, ChABC + polyLM. The two medications in each group were delivered intraspinally (see details below) separated by an interval of 1 week.

**Figure 1:**
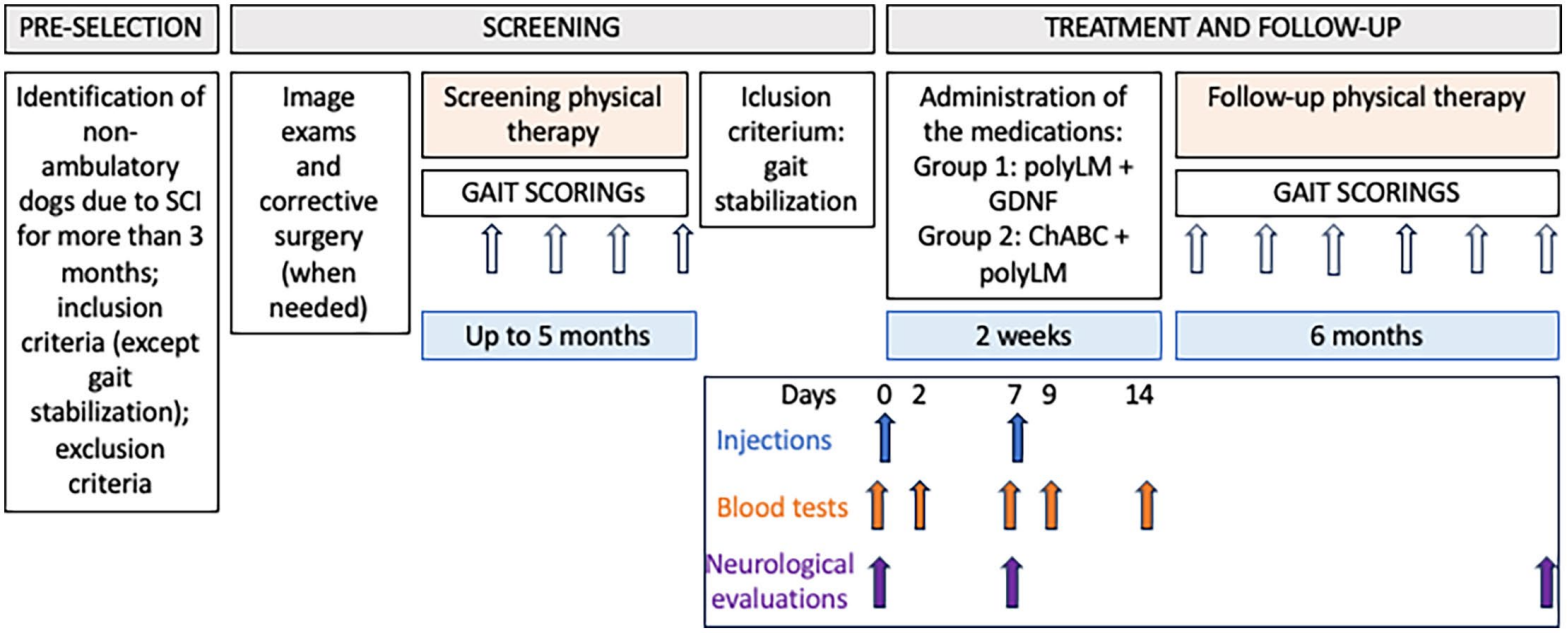
Flow chart of the study.

The follow-up lasted for 6 months and comprised neurological examinations and blood sample collections at 0, 2, 7, 9, and 14 days for the safety outcome (see details below), monthly gait evaluations (once per month), in addition to the continuation of the same physiotherapy program used along the screening. At the end of the study participants received a final neurological examination. The flow chart of the study is presented in Figure 1.

### 2.2 Inclusion and exclusion criteria

The study accepted non-ambulatory dogs with chronic SCI with lesions localized between T3 and L3. A minimum of 3 months between loss of ambulation and the initiation of screening physical therapy was required. Animals with SCI resulting from accidental trauma and intervertebral disc degeneration were included. The definitive criterion for inclusion was obtaining three stabilized scores during the screening physical therapy. Only animals with extremely severe spinal cord injuries or orthopedic conditions in the limbs that would preclude gait recuperation, as assessed by the study veterinarians, were excluded. Inclusion and exclusion criteria were applied at the beginning of the screening, except for the evaluation of gait stabilization.

### 2.3 Study medications

Polylaminin was used in a dose of 1 μg/kg of body weight. The polymerized protein at a final concentration of 100 μg/mL was prepared freshly at the surgery site by mixing laminin (Cristália, Itapira-SP, Brazil; batch no. PL13/22) with vehicle (20 mM acetate buffer containing 1 mM CaCl_2_) in a 1:1 (v/v) proportion (11). The mixture was poured into a sterile wide-mouth vessel and collected into a 1 mL syringe with a 26G needle. Polylaminin was delivered into the spinal cord, through one or more intramedullary injections. The injection sites were previously defined based on the image exams of each animal so that the medication would be delivered into the preserved tissue as close as possible to the lesion epicenter and avoiding areas of malacia characterized by fluid-filled cavities. The identification of the targeted intervertebral spaces was initially performed through palpation. The surgeon then inserted the needle at the tentative site, without contacting the spinal cord. After iterative confirmation of the precise location via X-ray imaging, the needle was advanced into the cord and connected to the syringe containing the medication. Supplementary Figure S1 shows lesion images and illustrates the rationale behind the selection of injection sites for each individual dog. It is important to note that two dogs (P1 and P3) had MRI-incompatible materials in the vertebral column and, therefore, underwent CT myelography (CTM) instead of MRI. As a result, precise localization of the spinal cord lesions was not possible, and injection sites were determined based on the locations of vertebral fractures and contrast diffusion patterns (Supplementary Figure S1).

ChABC was used at a fixed dose of 5 U per dog. The enzyme was purchased lyophilized from AMSBIO (cat. no. AMS. E1028-10) and reconstituted to a concentration of 12.5 U/mL in 1 M trehalose for thermal stabilization (14, 22). A volume of 800 μl was administered intraspinally using the same procedure as done for polyLM.

Human GDNF was used at a fixed dose of 50 μg per dog. The factor was purchased from Peprotech (cat. no. 450–10) and reconstituted in saline to a final concentration of 250 μg/ml. A volume of 200 μl was administered intraspinally as done for polyLM, except for the injection site, which was defined as the next intervertebral disc space caudal to the polyLM injection site, according to the rationale proposed in previous studies (16, 17).

### 2.4 Safety assessment

Safety was assessed based on 3 evaluations. First, we compared neurological examinations performed before each injection with the final examination. Treatments were considered safe if no deterioration was observed during the follow-up. The second assessment was monitoring of medical complications throughout the follow-up, defined as all clinical events reported by the owners. Treatments were considered safe if no severe events were reported and if no mild or moderate events occurring during the follow-up could be attributed to the medications. The third safety assessment was the measurement of serum markers of renal and hepatic damage (urea, creatinine, AST, ALT, total serum proteins and alkaline phosphatase) at days 0, 2, 7, 9, and 14 of the follow-up. Treatments were considered safe if no trend toward elevation in comparison with the initial values (before treatment) were observed and if no moderate or severe deviations from reference values were detected.

### 2.5 Gait analysis

A possible effect of the treatments in improving motor function was explored by comparing gait performances before and after treatment. Performance was graded using two previously validated gait scales, the TSCIS (23) and the OFS (24). Animals were videotaped once per month walking on a non-slip surface guided by a leach, with and without weight support. Two independent raters trained using a previously validated training module attributed the scores (25). Since the present study was single-arm and open-label, meaning that both the study team and the dog owners knew about the administered treatments, any observed improvement of gait function could not be considered as a definitive demonstration of efficacy.

### 2.6 Statistical analysis

The effect of polyLM treatment was estimated using a linear mixed model, with random intercepts for the participant ID and the experimenter, as well as a fixed effect for the intervention indicator. This modeling approach can account for individual changes between scores at baseline and end of study, as well as determine the effect of the intervention while adjusting for other covariates. This model was used as the basis for a preliminary efficacy analysis, determining the average effect of the treatment during the study period between the baseline and the follow-up periods. This estimate was adjusted by the secondary treatment to estimate the comparative effect of GDNF over ChABC. Other covariates were included to further adjust the effect estimate as study power allowed, included in the model following the order: secondary treatment, experimenter, sex, age, multiple lesions, recent lesions and breed. Each score (TSCIS and OFS) was treated separately, but both using the same model specification.

A second model was fit to explore if the rate of changes in the motor functional scores varied over time. To include the temporal dimension explicitly in the model a random slope for the session indicator was added to the participant id random intercept together with a fixed effect interaction between the session and the treatment indicator. This allowed for the rates of changes to vary independently over time providing a complementary analysis to estimate the cumulative effect of time on the outcome.

The validation of experimenter variability was performed following a fully crossed factorial design, where the factors subject id, session indicator and experimenter are modeled as random effects. The variance observed for each factor was extracted from the model and the total variance was calculated. The variance of the experimenter condition was reported as a proportion of the total variance in the experiment.

All analyses were performed using the significance level of 5%. All significance hypothesis tests, and confidence intervals computed were two-tailed. The analysis was performed using statistical software R version 4.3.0. The complete analytical plan is provided as Supplementary material.

## 3 Results

### 3.1 Screening period

Eight non-ambulatory animals were pre-selected for the screening program based on their clinical examinations and imaging exams, location of the injury (T3/L3) and historical records obtained during the interview with the owners. The owners signed a written consent for participation in the project. Three of these 8 dogs had already received decompressive/stabilization surgery when they entered the project and immediately initiated the physiotherapy of the screening (aiming to register their baseline gait before the experimental intervention). Five dogs had no previous surgery and were submitted to spinal decompression and pediculectomy before initiation of the screening physiotherapy. The duration of the screening physiotherapy ranged between 4 and 5 months depending on each animal’s response. Pre-selected dogs PRE-7 and PRE-8 kept the initial gait scores stabilized along the whole duration of the screening and were selected for inclusion in the clinical trial, becoming participants P3 and P6, respectively (Table 1). PRE-1, PRE-3, PRE-5, and PRE-6 experienced gait score improvements in the first month of the screening physiotherapy but the progress stabilized from the first month on. They were included in the trial, receiving the numbers P1, P4, P2 and P5, respectively. Dog PRE-4 had a progressive increase in motor function (gait scores increased from grades 2 to 4 in both scales) within the first 2 months of the screening and was excluded. Dog PRE-2 died suddenly due to suspected leptospirosis on January 27, 2023.

**Table 1:** Characteristics of the study subjects.

Injected volumes	Pre-1	Pre-2	Pre-3	Pre-4	Pre-5	Pre-6	Pre-7	Pre-8
ID Number	P1	-	P4	-	P2	P5	P3	P6
Dog breed	Mixed-breed	Poodle	Poodle	Dachshund	Bulldog	Shih Tzu	Mixed-breed	Mixed-breed
Sex	M	M	M	F	F	M	F	F
Age	3 yo	6 yo	4 yo	4 yo	9 yo	4 yo	6 yo	7 yo
Cause of injury	Run-over injury	Disk degeneration	Disk degeneration	Disk degeneration	Disk degeneration	Disk degeneration	Run-over injury	Disk degeneration
injury level	(T8-9) T10 to T12	L2 to L3	T13 to T13-L1 L3	T11-T12 T13-L1 L3-L4	L1 to L1-2	T10-11 to T12-13	(T3-4) T10 to T11	L2-3 to L4-5
Time of lesion^1^	6 months	3 months	4 months	6 months	7 years	11 months	5 years	4 months
Consent to participate^2^	09-02-2022	09-02-2022	09-03-2022	09-03-2022	09-05-2022	09-14-2022	09-17-2022	09-24-2022
Previous surgery	Arthrodesis	–	–	–	–	Decompression	Arthrodesis	-
Surgery within the project	–	Decompression pediculectomy	Decompression pediculectomy	Decompression pediculectomy	Decompression pediculectomy	-	-	Decompression Pediculectomy
Screening initiation^3^	09-23-2022	11-12-2022	11-12-2022	12-10-2022	10-07-2022	11-12-2022	10-17-2022	11-14-2022
Duration of the screening^4^	5 months	-	4 months	-	4.5 months	4 months	4 months	5 months

1- Refers to the time between loss of ambulation and beginning of screening physiotherapy; 2- Written consent to participate in the project; 3- Beginning of screening physiotherapy; 4- Total time in screening physiotherapy to reach stability of gait scores (3 consecutive scores varying no more than 1 point).

### 3.2 Demography of the study

The clinical trial proper included a cohort of 6 animals. They were 3 males and 3 females (50% males), aged 3 to 9 years (mean age = 5.5 ± 2). Three were mixed breed, one was a French Bulldog, one a Poodle, and one a Shih Tzu. The causes of injury were intervertebral disc degeneration (4) or accidental run-over injuries (2). The time elapsed between the loss of ambulation and the time when the animals started receiving the screening physiotherapy varied between 4 months and 7 years (mean time = 28.2 ± 31 months), whereas 2 of them (33.3%) were paralyzed for more than 1 year. Half of them (3) had received decompression/stabilization surgery at the time of ambulation loss and half received decompressive surgery as part of the project.

### 3.3 Treatments

The 6 animals selected to receive the experimental intervention (P1-P6) had their owners informed about the nature of the medication and the commitments involved in study participation. They signed the written consent for their dogs’ participation in the clinical study. The first three to reach stabilization were allocated to the first group, randomly chosen to receive polyLM and GDNF (group 1) and the next three were assigned to the group receiving ChABC and polyLM (group 2). The three participants in each group received the treatments on the same treatment dates (Table 2).

**Table 2:** Description of treatments.

			First Injection				Second Injection			
Experimental group	Patient	Weight	Date	Treatment	Location	Volume Injected	Date	Treatment	Location	Volume Injected
**Group 1** PolyLM (1 μg/kg) + GDNF (50 μg)	P1	9 kg	02–28-23	polyLM (100 μg/ml)	T8-9 T11-12	45 μl 45 μl	03-07-23	GDNF (250 mg/mL)	T9-10 T12-13	100 μL 100 μl
	P2	9 kg	02–28-23	polyLM (100 μg/mL)	T13-L1 L2-3	45 μl 45 μl	03-07-23	GDNF (250 mg/ml)	L3-4	200 μL
	P3	16 kg	02–28-23	polyLM (100 μg/ml)	T9-10	160 μl	03-07-23	GDNF (250 mg/ml)	T11-12	200 μl
**Group 2** ChABC (5 U) + PolyLM (1 μg/kg)	P4	11 kg	03–14-23	ChABC (12.5 U/mL)	T12-13 T13-L1 L1-2 L3-4	100 μl 100 μl 100 μl 100 μl	03-21-23	polyLM (100 μg/mL)	T12-13 T13-L1 L1-2 L3-4	30 μL 30 μL 30 μL 30 μL
	P5	8 kg	03–14-23	ChABC (12.5 U/mL)	T10-11 T12-13	200 μl 200 μl	03-21-23	polyLM (100 μg/mL)	T10-11 T12-13	40 μL 40 μL
	P6	9 kg	04–18-23	ChABC (12.5 U/mL)	L2-3 L4-5	200 μl 200 μl	04-25-23	polyLM (100 μg/mL)	L2-3 L4-5	45 μl 45 μL

On the day of the treatment the dogs were welcomed by the trial team and, before the procedure, they underwent a clinical/neurological examination, had blood samples collected and received gait evaluation. The gait scores assigned immediately before treatment corresponded to a fourth value, which, together with the three previously stabilized gaits scores recorded during the screening, comprised the gait baseline before treatment for each animal.

The first injections in the two groups consisted of polyLM (group 1) or ChABC (group 2). According to the protocol, both treatments should be injected as close as possible to the injury site, avoiding areas of frank malacia. The sites were defined individually for each animal after evaluation of the imaging exams (Supplementary Figure S1). The second injections were of GDNF (group 1) or polyLM (group 2). As the rationale of using GDNF is to attract growing axons towards a distal region of the spinal cord (16), it was injected at the next intervertebral space caudal to the lesion, while again avoiding areas of malacia. The injection of polyLM in group 2 was performed at the same sites where ChABC was delivered. It is important to note that in two dogs (P1 and P3), MRI could not be performed to accurately localize the lesions within the spinal cord. As a result, injection sites in these cases were determined based solely on CT myelography (Supplementary Figure S1). We acknowledge that the absence of MRI data in these animals represents a limitation of our study.

The injections were performed without any complications and as planned. Dogs recovered from anesthesia and remained under observation for 1 h, after which they were returned to their owners.

### 3.4 Safety assessment

Safety was assessed by comparing neurological examinations before and after the injections, monitoring of adverse events during the follow-up, and analyses of serum markers of hepatic and renal damage. Animals received three clinical/neurological evaluations, two immediately before each injection and one at the end of the study. The results are compiled in Supplementary Figure S2, which shows that in the beginning all participants had preserved arc reflexes, with reduced intensities observed only for patellar reflex in dogs P4 and P6 and perineal reflex in dog P4. None of the participants presented proprioception or pain perception. At the end of the follow-up the only deterioration observed was a reduction of the femoral reflex in the right hind limb of dog P3. A notable finding was that dog P3 recovered deep pain sensitivity at the final examination.

The only medical complication reported by the owners was an episode of diarrhea in dog P5 occurring on the ninth day of the follow-up, which corresponded to day 2 after the second injection (in group 2, the second injection was polyLM). The animal received a prescription for an anthelmintic, a probiotic, and a vitamin supplement. The symptoms disappeared after 24 h. No other medical events were reported by the owners.

**Figure 2:**
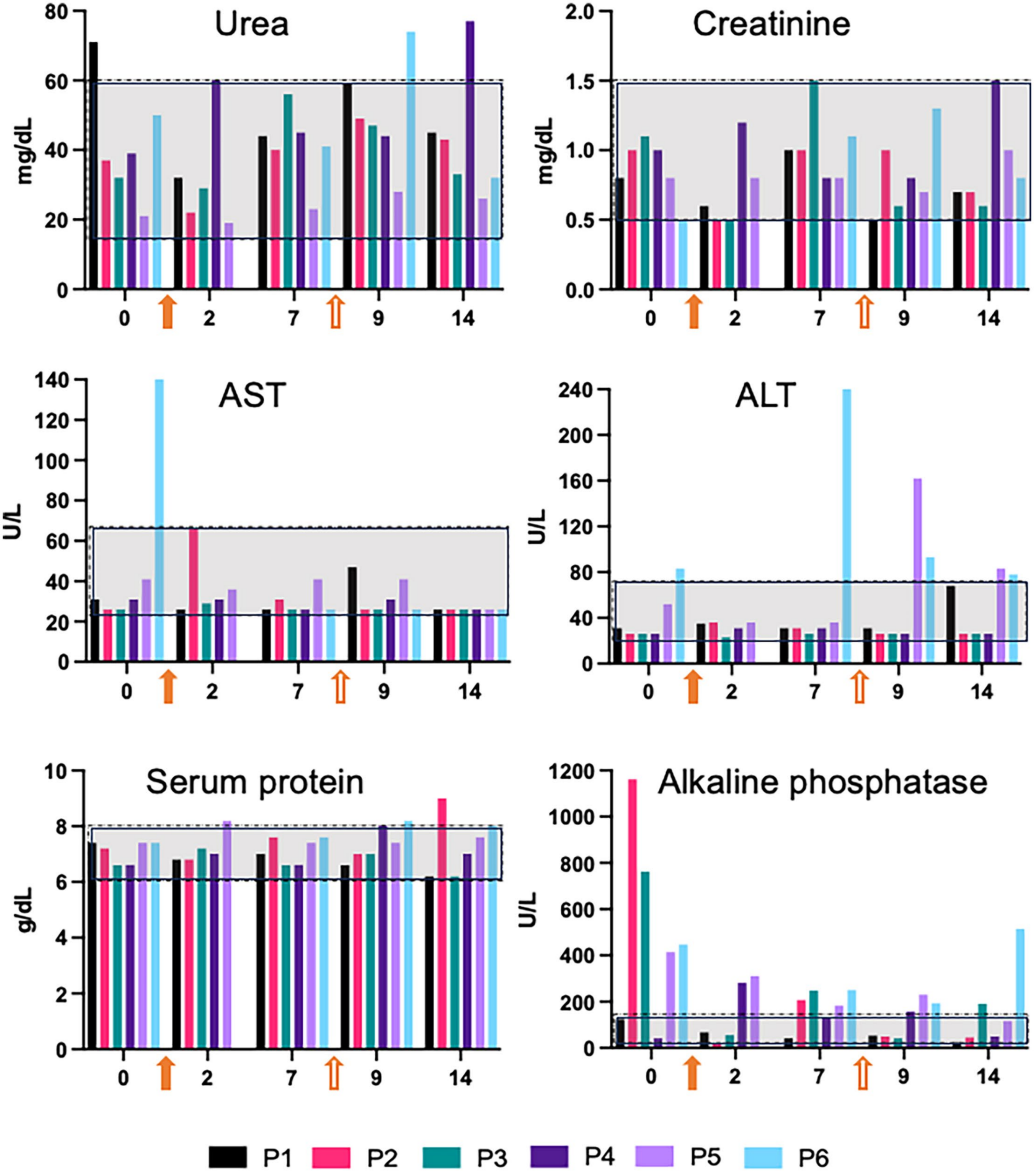
Serum markers of kidney and liver toxicity. Blood tests were carried out before the injections and on days 2, 7, 9, and 14 of the follow-up. On day 7 blood samples were collected before the second injection, meaning that this time point corresponds both to day 7 after the first injection and to day 0 of the second. Reference ranges are indicated by gray rectangles. The filled and empty orange arrows represent the first and second injections, respectively. Note that for dogs P1-P3, the first injection was polyLM and the second, GDNF. For dogs P4-P6, the first and second injections were ChABC and polyLM, respectively.

To investigate a possible toxic effect of the medications, we measured serum markers of kidney and liver damage before and after the injections (Figure 2). Serum levels of creatinine remained within the reference range throughout the measurements at 0, 2, 7, 9 and 14 days. The same occurred to urea, except for two mild elevations observed on the final exam of dog P4 and on the fifth exam of P5. As for hepatic toxicity, AST levels after the injections stayed within the reference limits across the evaluation period. In dog P5 we found a mild elevation of ALT on the 9^th^ day, i.e., the second day after the second injection (in group 2 the second injection was polyLM). The result of this blood test was released on the same day of the diarrhea episode reported above and, since there was a concomitant elevation in band neutrophils in the hemogram, it is likely that the animal experienced an infectious gastrointestinal event. ALT levels in dog P5 decreased to near normal on the following exam. Dog P6 had a peak of ALT on day 7, but this was before the second injection. Moreover, the animal had an ALT level above the reference limit even before the injections. Levels of total protein did not show notable alterations. Except for dog P1, the other five participants presented some degree of elevation in alkaline phosphatase levels. Such elevations however mostly correlated to higher levels of the enzyme already observed before the injections.

### 3.5 Analyses of gait performance

**Figure 3:**
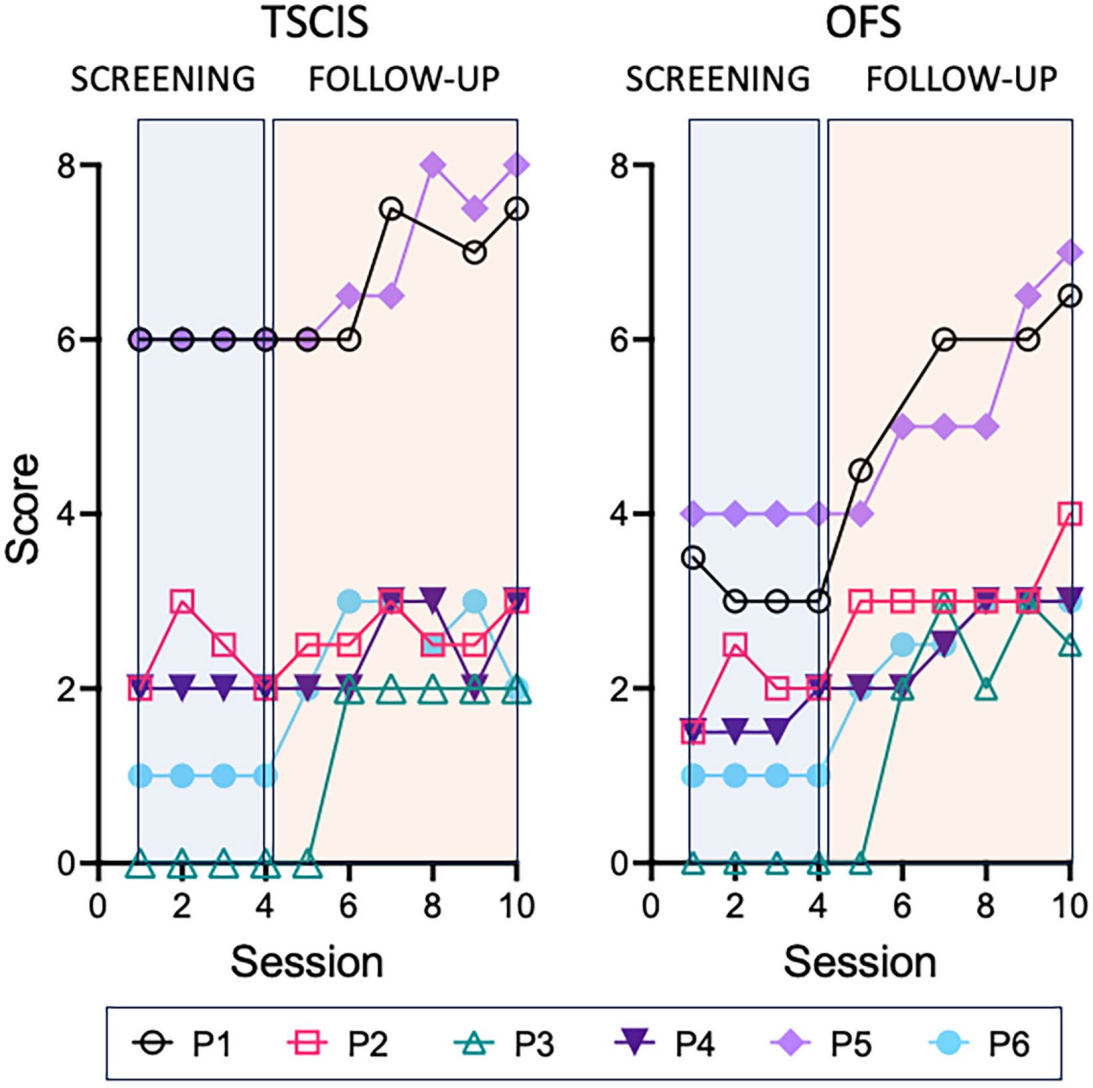
Gait scores across the whole study (raw data). Animals were evaluated using either the TSCIS or OFS gait scales along 4 sessions during the screening period (blue rectangle) and 6 sessions during the follow-up (orange rectangle). Each value corresponds to the mean of the scores attributed by two independent examiners. Two OFS scores and one TSCIS were discarded due to the use of a chest collar during the evaluation.

Two weeks after the second injection, dogs returned to the same twice-a-week physiotherapy routine implemented during the screening. They were evaluated using the TSCIS and OFS scales once a month for 6 consecutive months. The scores obtained for the six animals are shown in Figure 3. In both scales the follow-up scores of dogs P1, P3, P5, and P6 showed a clear trend of improvement compared to the screening (baseline) scores. Animals P2 and P4 also experienced a subtle improvement, more evident in the OFS scale.

### 3.6 Statistical analysis of gait scores

In total, 118 TSCIS observations and 114 OFS observations were available for analysis. Table 3 shows the estimated effect of the combination treatments for each metric. On average, both scores showed an increase during the study period. The estimated TSCIS score at the screening was 2.2, when assuming reference levels for all variables included in the model. At the follow-up the TSCIS score had an average increase of 0.99 (95% CI: 0.77, 1.2; *p* < 0.001) points. After accounting for all other variables, the estimated OFS score at follow-up was 1.5 (assuming reference conditions). At the follow-up the OFS score had an average increase of 1.6 (95% CI: 1.3, 1.9; p < 0.001) points in the scale.

**Table 3:** Effect of the treatment on TSCIS and OFS scores.

Characteristic	*N*	Beta^1^	95% CI^2^	*p*-value
TSCIS	118	0.99	0.77 to 1.2	<0.001
OFS	114	1.6	1.3 to 1.9	<0.001

^1^ Adjusted for secundary treatment, experimenter, sex and multiple lesions. ^2^ CI: Confidence Interval.

**Figure 4:**
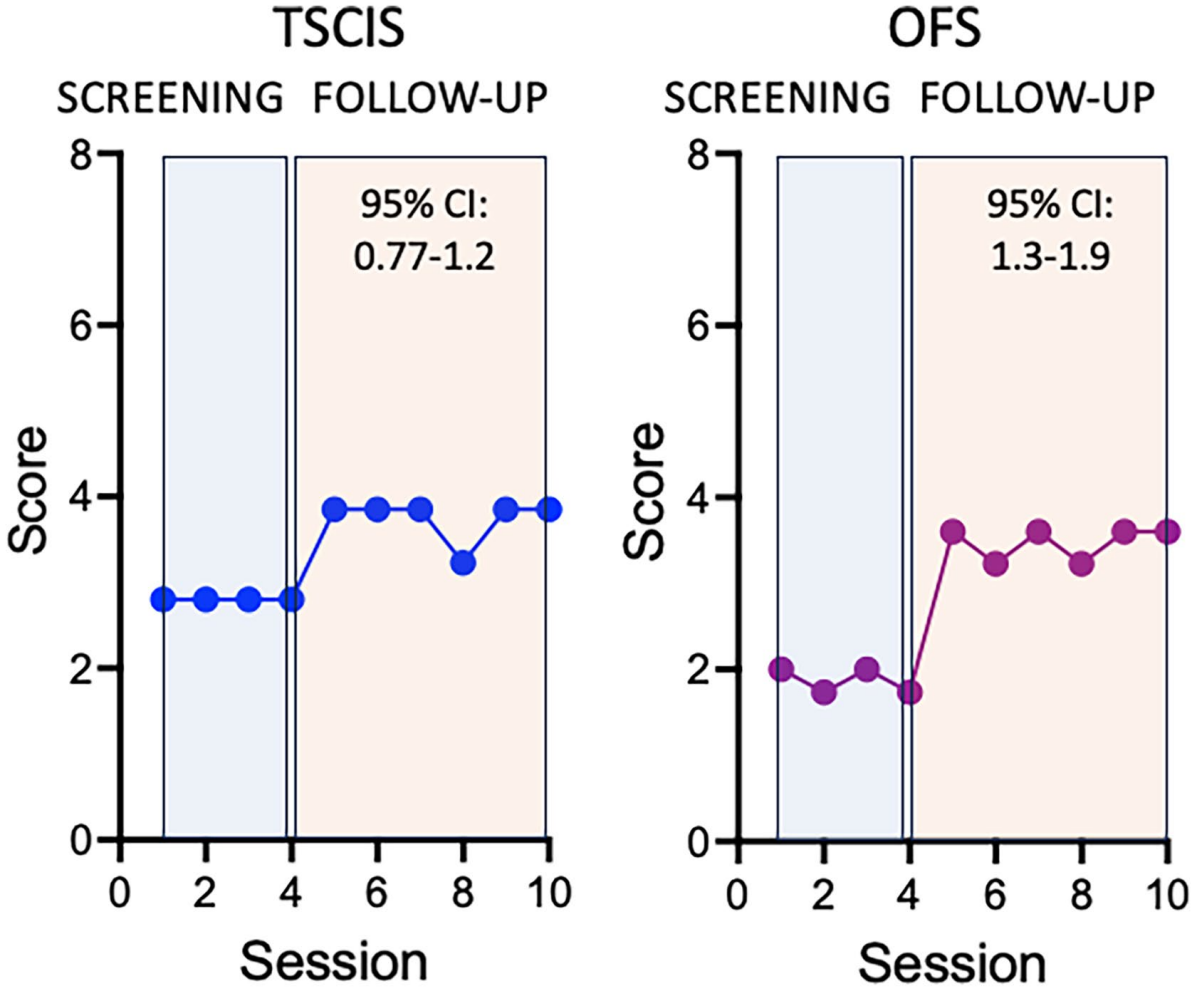
Time-varying effect of the treatment on TSCIS and OFS scores. Values were calculated per session after adjusting for secondary treatment, experimenter, sex, and multiple lesions. Here the session was used as a proxy of the exact amount of time elapsed between observations. This is because the subjects were followed-up at regular intervals and small delays between consecutive sessions were not sufficient to produce bias in the analysis.

Figure 4 shows the progression of average scores during the study period in both metrics. Both TSCIS and OFS scores show, on average, a significant increase after the intervention was applied. These data provide evidence of improvement when considering the average effect over the study period.

The effects of polyLM + GDNF and ChABC + polyLM do not appear to differ from one another. Assuming ChABC + polyLM as the reference, the 95% CI of the difference is [−9.98, 9.54] for the TSCIS score and [−6.84, 6.30] for the OFS score. Both 95% CIs cross the null hypothesis threshold and are wide enough to indicate considerable uncertainty around the estimate of the differences in effects between them. Therefore, there is no evidence of a differential effect between the two experimental groups.

For each metric, a second model was fit to the data including only random effects for the design terms (subject ID, session indicator and experimenter). This pair of models can be used to calculate the variance of each experimental condition (Table 4). In both cases, most of the variance observed arises from the subjects. In the case of the TSCIS the variance due to the experimenter as a proportion of the total variance in the experiment was <0.1%. The variance of the OFS was 0.2% of the total variance. These are minute relative variances and demonstrate high precision across personnel and consistency of measurements over time and between study subjects. There is no evidence that introducing a third experimenter would have a substantial impact in the measurements.

**Table 4:** Variance due to each experimental condition and total variance observed.

Metric	id	Session	Experimenter	Residual	Total
TSCIS	5.9909	0.3589	0.0026	0.2606	6.6130
OFS	2.1635	0.8646	0.0077	0.2737	3.3095

**Figure 5:**
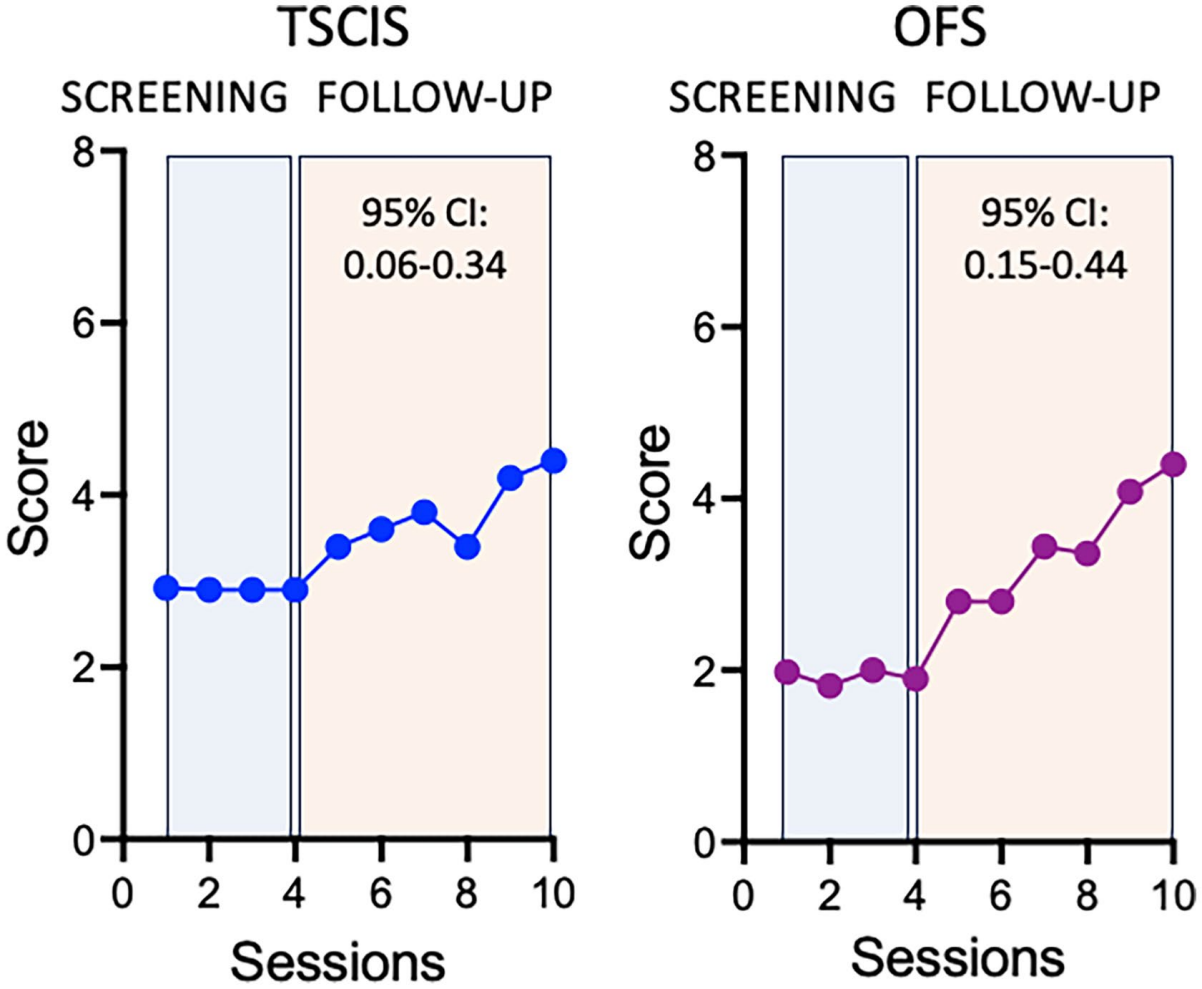
Average effect of the treatment on TSCIS and OFS scores. Average values were calculated per session after adjusting for secondary treatment, experimenter, sex and multiple lesions.

We next describe a supplemental approach that allows the effect to vary over time, thereby estimating the increment in functional scores per follow-up session after the intervention. This was achieved by including an interaction term between the treatment and the session indicator. When such interaction terms are significant, it is customary to disregard the interpretation of the individual terms and focus instead on the interaction itself. Figure 5 shows how the incremental increase impacts the average score on both TSCIS and OFS scales.

The interaction between the treatment and the session was significant for the TSCIS scale with an increase of 0.20 (95% CI: 0.06, 0.34; *p* = 0.005) points per session following the intervention. With all other variables kept constant, this means that after 5 follow-up sessions one could expect an increase of one point on the TSCIS scale, after adjusting for secondary treatment, sex, and multiple lesions. The interaction was also significant in the OFS analysis. After treatment, there was an incremental increase of 0.30 (95% CI: 0.15, 0.44; *p* < 0.001) points per session, after adjusting for the other variables. A one-point increase in this scale could be expected after four post-intervention sessions. The intercepts of these models are reported in Table 5. A complete description of the statistical analysis is provided as Supplementary material.

**Table 5:** Coefficients for both efficacy analyses.

	TSCIS average effect			OFS average effect			TSCIS time-varying effect			OFS time-varying effect		
Characteristic	Beta	95% CI^1^	*p*-value	Beta	95% CI^1^	*p*-value	Beta	95% CI^1^	*p*-value	Beta	95% CI^1^	*p*-value
(Intercept)	2.2	−5.7 to 10	0.349	1.5	−3.8 to 6.8	0.349	5.2	−2.8 to 13	0.112	1.5	−3.6 to 6.7	0.344
Treatment	0.99	0.77 to 1.2	<0.001	1.6	1.3 to 1.9	<0.001	−0.49	−1.1 to 0.11	0.110	−0.74	−1.4 to −0.13	0.018
Secundary treatment												
Chondroitinase ABC	–	–		–	–		–	–		–		
GDNF	0.22	−10 to 9.5	0.932	−0.27	−6.8 to 6.3	0.877	3.4	−4.6 to 11	0.210	−0.26	−6.8 to 6.3	0.881
Sex												
F	–	–		–	–		–	–		–	–	
M	2.9	−6.8 to 13	0.327	1.8	−4.8 to 8.4	0.356	−0.75	−8.7 to 7.2	0.723	1.8	−4.8 to 8.3	0.366
Multiple lesions	−1.4	−11 to 8.4	0.600	−0.60	−7.2 to 6.0	0.733	−7.2	−15 to 0.81	0.061	−0.77	−7.3 to 5.8	0.662
Sessions							−0.01	−0.15 to 0.13	0.907	0.03	−0.12 to 0.17	0.710
Treatment Sessions							0.20	0.06 to 0.34	0.005	0.30	0.15 to 0.44	<0.001

^1^CI: Confidence Interval.

## 4 Discussion

Despite decades of intensive scientific efforts to develop therapies capable of reversing the paralysis caused by spinal cord injuries, an effective treatment is still an unmet need for both animals and humans. It is currently understood that an effective treatment for spinal cord injury will require the combined use of several therapeutic approaches (26), which together may restore the ability of neurons to extend axons through an environment that is particularly non-permissive to regeneration. In recent years, our laboratory has been working on the development of polyLM to be used as a therapeutic agent for treating spinal cord injuries. This biotechnological product is based on the natural protein laminin, which plays multiple roles during the development and regeneration of the nervous system. The fact that it exhibits multiple functions, and all favoring neural plasticity, suggests that polyLM therapy might, by itself, act as a multi-action drug. This interpretation is consistent with the positive results of a pilot clinical study in which we tested the safety and potential therapeutic effect of the injection of polyLM in human patients during the acute phase of SCI (the first days after trauma). In that study, we observed that patients with complete injury—that is, those with minimal chances of recovering movement below the level of the lesion—regained motor control within the first months following treatment. In the present study, we aimed at a proof of concept that polyLM could be used to treat SCI even in the chronic phase, despite the presence of an established non-permissive scar at the lesion site. We found that polyLM combined with either ChABC, to digest inhibitory chondroitin sulfate proteoglycans present in the scar, or GDNF, a chemoattractant to growth cones, was a safe and effective approach to treat chronic SCI in dogs. The significance of our findings, as well as their limitations will be discussed in this section.

Our safety assessment demonstrated no medical complications, no neurological deterioration and no clinically significant changes in blood tests following treatment in 5 out of 6 dogs. Only dog P5 presented an episode of diarrhea, which occurred 2 days after receiving polyLM. The event was concomitant with an increase in ALT, suggesting that it could be related to a gastrointestinal infection causing hepatotoxicity. Alternatively, the two findings could be unrelated or possibly associated to the administration of anesthetics used for sedation. Both the clinical symptoms and the ALT elevation resolved rapidly. In principle, we could not rule out the possibility that these events were related to polyLM administration. However, the fact that the other five animals did not present any of these features after polyLM injection makes this interpretation unlikely. Moreover, polyLM had been administered to human patients in a previous clinical trial and no liver toxicity was suspected (11). Finally, a contract research organization (CRO) conducted a study in rats to evaluate the potential toxicity of intramedullary injection of polyLM. No signs of hepatic damage were observed, as evidenced by the absence of alterations in serum markers and in the histopathological analysis of the liver.

Notably, we found elevations in alkaline phosphatase in all but one dog (P1). Dogs P2, P3, P5 and P6 had at least one event of mild to moderate elevation during the follow-up. Nevertheless, the levels above the reference limits were never higher than those observed before the injections (time 0). Although alkaline phosphatase was used in the present study as a marker of hepatic damage, it is an enzyme present in other tissues, and its elevation may be related to several biological processes, such as bone mineralization. It is generally accepted that an isolated increase in serum levels of alkaline phosphatase without clinical manifestations is nonspecific and cannot be correlated to liver damage (27). One possible explanation for such findings is that these dogs (except P3) had IVDD and were subjected to a physiotherapy program that might have accelerated vertebral bone remodeling. Notably, physiotherapy was interrupted for 3 weeks during the injection period, which may have accounted for a general decrease in the serum levels over the 14 days of the blood tests. Only dog P4 experienced an increase in alkaline phosphatase relative to the value found at time 0. This occurred 2 days after ChABC injection. The elevation was mild, and the value returned to the normal range in the following evaluations.

Given that the injections did not result in any detectable neurological deterioration, that no adverse clinical events were reported during the follow-up, and that the evaluation of serum markers for renal and hepatic toxicity did not indicate damage to these organs, we conclude that the two combination treatments investigated in this study appear to be safe for dogs.

The primary outcome used to assess potential treatment effects was an increase in locomotion measured by two different scales, TSCIS and OFS, both of which exclusively evaluates the dog’s gait performance. Gait scales are commonly employed in clinical trials because they permit the objective quantification of recovery, while providing a sensitive tool to probe incremental improvements of a relevant function. The reason for using two scales instead of only one, relates to the fact that each one focuses on slightly different aspects of gait. Both have 12 grades, but the TSCIS analyzes each hindlimb separately and is more adequate to evaluate lateralized lesions. On the other hand, the OFS analyzes both hindlimbs together but is more sensitive to detect subtle improvements in locomotion, particularly in ambulatory dogs (25). Our study was an exploratory trial with no previous hypothesis and inclusion criteria that were deliberately broad, encompassing animals with SCI caused by accidental trauma and IVDD. We thus predicted that the cohort of animals enrolled would comprise a great variability of lesions better addressed with the use of two complementary scales. Indeed, we ended up with a sample of 6 animals, with 2 having accidental trauma and 4 having IVDD with variable degrees of severity. We could detect significant improvements after treatment using both scales. Nonetheless, the magnitude of the effects was more pronounced when the OFS was used, which probably reflects the fact that the gait improvements observed following treatment, although statistically significant, were relatively modest and required a more sensitive scale.

One important limitation of our study was that we tested two combination treatments but did not test the effect of each individual treatment separately. When all data obtained for each metric (TSCIS or OFS) were pooled into a single statistical analysis (that considers the hierarchization of data), it was possible to demonstrate the effect of the treatments. However, there was no detectable distinction between the two groups. The lack of clear superiority between the two combination treatments allows for multiple interpretations. First, the two secondary treatments, ChABC and GDNF, may contribute equally to the potential benefits of polyLM. Second, polyLM alone could be responsible for the observed gait improvement, a hypothesis supported by previous findings showing that contact with endogenous laminin can render axons insensitive to inhibitory chondroitin sulfate proteoglycans (16). A third possibility is that polyLM was ineffective, and that gait improvement in our study resulted primarily from the individual effects of GDNF (group 1) and ChABC (group 2), both of which have been shown to promote functional recovery in experimental models of SCI (15, 17, 28). Additionally, prior studies have reported positive effects of ChABC treatment in dogs with SCI (14, 29). Finally, a fourth interpretation is that none of the treatments was effective, and that the observed improvement was due to the injection procedure itself, which could have induced local inflammation and promoted a regenerative response. To distinguish among these possibilities, future studies will need to include additional experimental groups, such as a placebo group (vehicle injection only) and groups receiving each treatment individually.

Another limitation of the present study was the variability within the sample, which precludes extracting information about a possible correlation between one given characteristic (e.g., sex, age, breed, type of lesion, lesion size) and the magnitude of the benefit associated to the medication. Accordingly, we could not identify a subgroup of individuals more prone to a favorable outcome. A possible exception perhaps was the observation that the animals that presented the most pronounced trend to gait improvement (P1, P3 and P5) were those who received decompression surgery within the first days after losing ambulation. Dogs P1 and P3 had accidental injuries that required immediate surgical intervention for stabilization of the vertebral column and dog P5, whose owners had the financial means to arrange early surgery. The three dogs that received decompression of the spinal cord, which was provided by the project months after loss of ambulation had the least pronounced recuperation (P2, P4 and P6). This interpretation is in line with a recent trend in the literature supporting the notion that early and effective decompression is a key factor to improve the patient’s prognosis after SCI. On that account, the beneficial effect of early surgery after SCI in humans has grown in evidence (30). In addition, there was a veterinary trial conducted in dogs indicating that the effect of cell therapy was more effective when associated to early surgery (31). Lastly, the hypothesis that improving spinal decompression by associating bone removal with duroplasty may improve the outcome of human SCI is the object of a promising current clinical investigation (32).

In this study, we reported that treating dogs with chronic spinal cord injuries using a combination of polyLM with either GDNF or ChABC was safe and associated with improvements in gait performance. However, the small and heterogeneous nature of our cohort limited our ability to distinguish the individual contributions of polyLM, GDNF, and ChABC to the observed outcomes. Moreover, the absence of a placebo group precludes definitive conclusions regarding treatment efficacy. Nevertheless, we believe that our findings offer promising insights and underscore the importance of conducting larger, more comprehensive clinical trials to further develop effective therapies aimed at reversing paralysis in both dogs and humans.

## Data Availability

The raw data supporting the conclusions of this article will be made available by the authors, without undue reservation.
