# Supplementary material for: A laminin-based therapy for dogs with chronic spinal cord injury: promising results of a longitudinal trial
Source: Front Vet Sci. 2025 Aug 13;12:1592687. doi: 10.3389/fvets.2025.1592687 (PMC12380836; doi:10.3389/fvets.2025.1592687)
Supplement: Supplementary file 1 [file Data_Sheet_1.pdf]

Analytical Plan for Efficacy of polylaminin on the recovery of motor function after spinal cord injury: non-clinical trial

DOCUMENT: SAP-2023-034-TS-v02

From: Felipe Figueiredo **To:** Tatiana Sampaio

2023-12-28

TABLE OF CONTENTS

1 ABBREVIATIONS.....2
2 CONTEXT.....2
2.1 Objectives.....2
2.2 Hypotheses.....2
3 DATA.....3
3.1 Raw data.....3
3.2 Analytical dataset.....3
4 STUDY PARAMETERS.....3
4.1 Study design.....3
4.2 Inclusion and exclusion criteria.....4
4.3 Exposures.....4
4.4 Outcomes.....4
4.5 Covariates.....4
5 STATISTICAL METHODS.....5
5.1 Statistical analyses.....5
5.1.1 Descriptive analyses.....5
5.1.2 Inferential analyses.....5
5.1.3 Statistical modeling.....5
5.1.4 Missing data.....6
5.2 Significance and Confidence Intervals.....6
5.3 Study size and Power.....6
5.4 Statistical packages.....6
6 OBSERVATIONS AND LIMITATIONS.....6
7 REFERENCES.....6
8 APPENDIX.....7
8.1 Availability.....7

Analytical Plan for Efficacy of polylaminin on the recovery of motor function after spinal cord injury: non-clinical trial

Document version

Version	Alterations
01	Initial version
02	New methodology for the experimenter validation separate from the main analysis was added to 5.1.3

1 ABBREVIATIONS

- CI: confidence interval
- GDNF: Glial cell line-derived neurotrophic factor
- OFS: Open Field Scale
- SD: standard deviation
- TSCIS: Texas Spinal Cord Injury Scale

2 CONTEXT

2.1 Objectives

1. To determine the efficacy of polylaminin on the recovery of motor function after spinal cord injury in dogs.
2. To determine the comparative efficacy between polylaminin with Chondroitinase ABC and polylaminin with GDNF
3. To determine the variance of functional scores measures due to switching experimenters

2.2 Hypotheses

1. Average functional scores are different after polylaminin is administered.
2. Chondroitinase ABC and GDNF have different efficacy profiles when applied together with polylaminin;
3. The variance due to the experimenters is small, relative to treatment effect sizes.

3 DATA

3.1 Raw data

The raw data was provided in two tables, one with the study subjects (N=6) and the measurements performed on them, where measurements from experimenters were encoded in separate columns. Both tables were merged into a single table that includes all observations and all characteristics by using the subject ID as the key. Sessions were recorded in video for later evaluation and outcome measurements, and the dates for the sessions were available in the original data.

The original data base had 14 variables collected on 60 observations.

3.2 Analytical dataset

A session indicator was created to account for the passing of time during the study. The treatment was coded as binary indicator after the last session of the baseline, to classify the measurements into before and after the drug administration. Measurements from experimenters were aggregated into a single variable per score (section 4.4).

After the cleaning process 14 variables were included in the analysis. The total number of observations excluded due to incompleteness and exclusion criteria will be reported in the analysis. Table 1 shows the structure of the analytical dataset.

Table 1 *Analytical dataset structure after variable selection and cleaning.*

id	exposure	ofs	tscis	group	date	session	experimenter	name	sex	age	race	lesion_multiple	lesion_recent
1													
2													
3													
...													
120													

All variables in the analytical set were labeled according to the raw data provided and values were labeled according to the data dictionary for the preparation of production-quality results tables and figures.

4 STUDY PARAMETERS

4.1 Study design

This is a non-clinical trial, performed in dogs, where all subjects were treated with primary treatment polylaminin and allocated to either GDNF or Chondroitinase ABC as a secondary treatment.

Subjects were followed-up for ten monthly sessions (Sampaio, 2023). The first 4 sessions occurred before the intervention under investigation was administered, and will be used to establish the baseline motor function.

Efficacy measure of effect for polylaminin is defined as the difference in the outcome measures (see section 4.4) between baseline and follow-up moments. Due to a lack of experimental control (placebo or otherwise) between Chondroitinase ABC and GNDF, those interventions will be compared against each other.

4.2 Inclusion and exclusion criteria

N/A

4.3 Exposures

This analysis will focus on the polylaminin treatment for the efficacy measure. The exposure will be encoded as an indicator after the first session the treatment was administered to the study subjects (section 3.2).

1. Polylaminin treatment

4.4 Outcomes

Specification of outcome measures (Zarin, 2011):

1. (Domain) Motor function
2. (Specific measurement) TSCIS and OFS scores
3. (Specific metric) Change from baseline
4. (Method of aggregation) Mean

Primary outcomes

1. Average change in TSCIS score from baseline
2. Average change in OFS score from baseline

4.5 Covariates

1. Secondary treatment
2. Experimenter
3. Sex
4. Age (years)
5. Multiple lesions
6. Recent lesion
7. Race

5 STATISTICAL METHODS

5.1 Statistical analyses

5.1.1 Descriptive analyses

The epidemiological profile of the study participants will be described. Demographic (age and sex) and clinical variables will be described as mean (SD) or as counts and proportions (%), as appropriate. The distributions of participants' characteristics will be summarized in tables and visualized in exploratory plots.

5.1.2 Inferential analyses

All inferential analyses will be performed in the statistical models (described in the next section).

5.1.3 Statistical modeling

The effect of the polylaminin treatment will be estimated using a linear mixed model, with random intercepts for the participant ID and the experimenter, as well as a fixed effect for the intervention indicator (section 4.3). This modeling approach is able to account for individual changes between scores at baseline and end of study, as well as determine the effect of the intervention while adjusting for other covariates. This model will be used as the basis for the efficacy analysis, determining the average effect of the treatment during the study period between the baseline and the follow-up periods. This estimate will be adjusted by the secondary treatment to estimate the comparative effect of GDNF over Chondroitinase ABC (see Observations). Other covariates will be included to further adjust the effect estimate as study power allows, included in the model following the order presented in section 4.5. Each score defined in section 4.4 will be treated separately, but both will be treated with the same model specification.

A second model will be fit to explore if the rate of changes in the motor functional scores vary over time. Two approaches will be attempted to include the temporal dimension explicitly in the model. A random slope for the session indicator will be added to the participant ID random intercept together with a fixed effect interaction between the session and the treatment indicator. If the first approach does not produce a convergent model, a second approach will be attempted by including the interaction term described above but without the random slope for the session. Both approaches allow for the rates of changes to vary independently over time, and the resulting selected model (if any) will be presented as a complementary analysis to estimate the cumulative effect of time on the outcome.

The validation of experimenter variability will be performed following a fully crossed factorial design, where the factors subject id, session indicator and experimenter will be modeled as random effects. The variance observed for each factor will be extracted

from the model and the total variance will be calculated. The variance of the experimenter condition will be reported as a proportion of the total variance in the experiment.

5.1.4 Missing data

No missing data imputation will be performed. All evaluations will be performed as complete case analyses. Missing data counts and proportions will be reported in tables.

5.2 Significance and Confidence Intervals

All analyses will be performed using the significance level of 5%. All significance hypothesis tests and confidence intervals computed will be two-tailed.

5.3 Study size and Power

N/A

5.4 Statistical packages

This analysis will be performed using statistical software R version 4.3.0.

6 OBSERVATIONS AND LIMITATIONS

No control for the primary outcome

The choice for study design includes two different secondary treatments but there were no control groups for polylaminin without those secondary treatments. This makes it hard to estimate the effect due to polylaminin alone. In this analysis the effect attributed to polylaminin also includes the effect of its conjugation with Chondroitinase ABC, as the reference level for the secondary treatment.

Recommended reporting guideline

The adoption of the EQUATOR network (http://www.equator-network.org/) reporting guidelines have seen increasing adoption by scientific journals. All clinical trials are recommended to be reported following the CONSORT guideline (Schulz K F, Altman D G, Moher D., 2010).

7 REFERENCES

- **SAR-2023-034-TS-v01** – Efficacy of polylaminin on the recovery of motor function after spinal cord injury: non-clinical trial
- Sampaio, TLC, (2023). ESTUDO CLÍNICO VETERINÁRIO DO EFEITO DA POLILAMININA ASSOCIADA À CONDROITINASE ABC OU AO FATOR

NEUROTRÓFICO DERIVADO DE GLIA EM LESÕES MEDULARES CRÔNICAS EM CÃES

- Zarin DA, et al. The ClinicalTrials.gov results database – update and key issues. N Engl J Med 2011;364:852-60 (https://doi.org/10.1056/NEJMsa1012065).
- Gamble C, et al. Guidelines for the Content of Statistical Analysis Plans in Clinical Trials. JAMA. 2017;318(23):2337–2343 (https://doi.org/10.1001/jama.2017.18556).
- Schulz K F, Altman D G, Moher D. CONSORT 2010 Statement: updated guidelines for reporting parallel group randomised trials BMJ 2010; 340 :c332 (https://doi.org/10.1136/bmj.c332).

8 APPENDIX

This document was elaborated following recommendations on the structure for Statistical Analysis Plans (Gamble, 2017) for better transparency and clarity.

8.1 Availability

The client has requested that this analysis be kept confidential until a future date, determined by the client. All documents from this consultation are therefore not published online and only the title and year of the analysis will be included in the consultant's Portfolio. After the agreed date is reached, the documents will be released.

The portfolio is available at:

https://philsf-biostat.github.io/SAR-2023-034-TS/
