# Supplementary material for: A laminin-based therapy for dogs with chronic spinal cord injury: promising results of a longitudinal trial
Source: Front Vet Sci. 2025 Aug 13;12:1592687. doi: 10.3389/fvets.2025.1592687 (PMC12380836; doi:10.3389/fvets.2025.1592687)
Supplement: Supplementary file 2 [file Data_Sheet_2.pdf]

Efficacy of polylaminin on the recovery of motor function after spinal cord injury: non-clinical trial

DOCUMENT: SAR-2023-034-TS-v02

From: Felipe Figueiredo **To:** Tatiana Sampaio

2024-03-13

TABLE OF CONTENTS

1 ABBREVIATIONS....2
2 CONTEXT....2
2.1 Objectives....2
3 METHODS....2
4 RESULTS....3
4.1 Study population and follow up....3
4.2 Effect of the treatment on TSCIS and OFS....5
4.3 Validation of experimenters....7
5 OBSERVATIONS AND LIMITATIONS....7
6 CONCLUSIONS....8
7 REFERENCES....8
8 APPENDIX....9
8.1 Exploratory data analysis....9
8.2 Modeling strategy....10
8.2.1 Model specification....10
8.2.2 Time-varying effect....11
8.2.3 Full listing of coefficients in all models....12
8.3 Availability....13
8.4 Analytical dataset....13

Efficacy of polylaminin on the recovery of motor function after spinal cord injury: non-clinical trial

Document version

Version	Alterations
01	Initial version
02	New graph of model effects (sections 4.2, 8.2.1)

1 ABBREVIATIONS

- CI: confidence interval
- GDNF: Glial cell line-derived neurotrophic factor
- OFS: Open Field Scale
- SD: standard deviation
- TSCIS: Texas Spinal Cord Injury Scale

2 CONTEXT

2.1 Objectives

1. To determine the efficacy of polylaminin on the recovery of motor function after spinal cord injury in dogs.
2. To determine the comparative efficacy between polylaminin with Chondroitinase ABC and polylaminin with GDNF
3. To determine the variance of functional scores measures due to switching experimenters

3 METHODS

The data procedures, design and analysis methods used in this report are fully described in the annex document **SAP-2023-034-TS-v01**.

This analysis was performed using statistical software R version 4.3.3.

4 RESULTS

4.1 Study population and follow up

A total of 6 subjects were included in the study, with 3 females (50%), average age 5.5 years and ranging between 3 and 9 years. Table 1 shows the characteristics of the study population by treatment group.

Table 1 *Subject characteristics in the study sample.*

**Characteristic**	**Chondroitinase ABC**, N = 3	**GDNF**, N = 3
**Sex, n (%)**		
F	2 (67%)	1 (33%)
M	1 (33%)	2 (67%)
**Age (years), Mean (SD)**	6.00 (3.00)	5.00 (1.73)
**Race, n (%)**		
Bulldog	1 (33%)	0 (0%)
Poodle	0 (0%)	1 (33%)
Shi tzu	0 (0%)	1 (33%)
SRD	2 (67%)	1 (33%)
**Multiple lesions, n (%)**	1 (33%)	2 (67%)
**Recent lesion, n (%)**	1 (33%)	3 (100%)

In total 60 functional scores measurements were made between 2022-12-05 and 2023-11-13 by two independent experimenters at each monthly session (Sampaio, 2023). After aggregating the observations from both experimenters there were N = 120 measurements available for analysis.

Figure 1 shows the raw scores observed during the study. It is worth noticing that, while measurements at the baseline present some variability, they are otherwise stable across

that period. There is no obvious trend showing either improvement or degradation of functional scores before the intervention is applied, and that facilitates the pre-post comparison in this analysis. During the baseline period the average TSCIS was 2.9 and the OFS was 1.9.

After the baseline period most individuals appear to show some increasing trend in both scores. We will investigate further the magnitude of the effect of the study treatments in the next section.

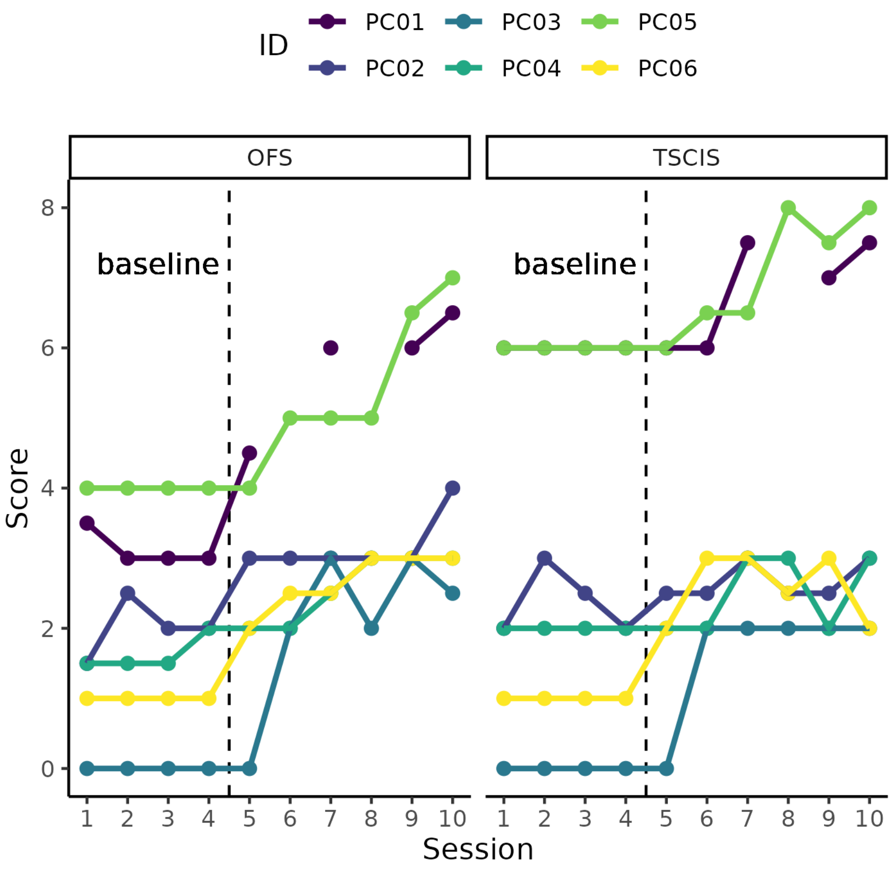

Figure 1 *Raw functional scores in the study sample.*

4.2 Effect of the treatment on TSCIS and OFS

In total, there were 118 TSCIS observations and 114 OFS observations available for analysis.

Figure 2 shows the marginal effects of the treatment during the study period in both metrics. Both TSCIS and OFS scores show, on average, an increase after the intervention is applied, after adjusting for secondary treatment, experimenter, sex and multiple lesions. An alternative visualization of the model estimates, showing the progression over time, is shown in the Appendix. A supplemental analysis that allows the effect to vary over time is available in the Appendix.

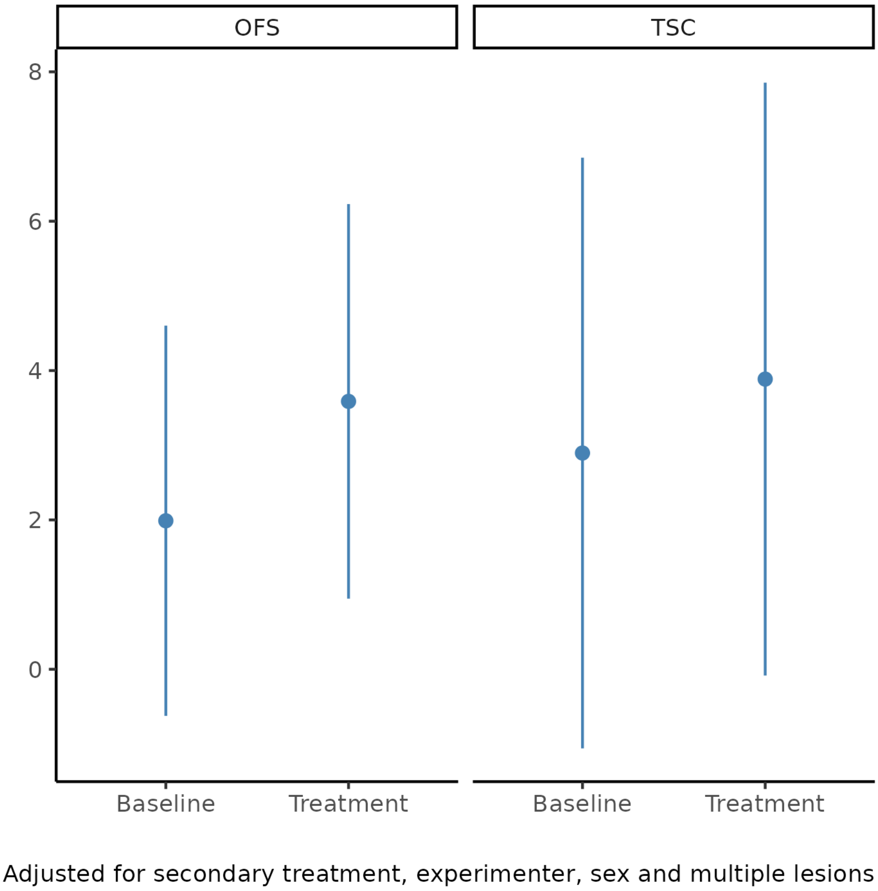

Figure 2 *Marginal effects of the treatment on TSCIS and OFS.*

Table 2 shows the estimates of the effect of the combination treatments for each metric. On average, both scores showed an increase during the study period. The basal estimated TSCIS score was 2.2, when assuming reference levels for all variables included in the model. This is the average TSCIS score after adjusting for the other variables and

assuming the following reference conditions: the secondary treatment being Chondroitinase ABC, sex being F and no multiple lesions). After the observation period the TSCIS score had an average increase of 0.99 (95% CI 0.77, 1.2; p<0.001) points in the scale.

After accounting for all other variables the basal estimated OFS score was 1.5 (assuming reference conditions). At the end of the observation period the OFS score had an average increase of 1.6 (95% CI 1.3, 1.9; p<0.001) points in the scale.

Table 2 *Effect of the treatment on TSCIS and OFS.*

**Characteristic**	**N**	**Beta**[1]	**95% CI**[2]	**p-value**
TSCIS	118	0.99	0.77 to 1.2	**<0.001**
OFS	114	1.6	1.3 to 1.9	**<0.001**

[1]Adjusted for secondary treatment, experimenter, sex and multiple lesions

[2]CI = Confidence Interval

The secondary treatments don’t appear to be different from one another. Assuming the Chondroitinase ABC as the reference, the 95% CI of difference is [-9.98, 9.54] for the TSCIS score and [-6.84, 6.30] for the OFS score. Both 95% CI cross the null hypothesis threshold (zero) and are wide enough that there is too much uncertainty around the estimate of differential efficacy between them. There is no evidence of a differential efficacy between both secondary treatments. Table A1 in the appendix shows the effects of all variables included in the analysis.

It’s worth noting that there was no option where the subjects received only the polylaminin intervention, so this analysis can only evaluate the primary and secondary treatments combined (see Observations and Limitations).

4.3 Validation of experimenters

For each metric a second model was fit to the data including only random effects for the design terms (subject id, session indicator and experimenter). This pair of models can be used to calculate the variance of each experimental condition (Table 3).

In both cases most of the variance observed arises from the subjects. In the case of the TSCIS the variance due to the experimenter as a proportion of the total variance in the experiment was <0.1%. The variance of the OFS was 0.2% of the total variance. These are minute relative variances and show high precision across personnel and consistency of measurements over time and between study subjects. There is no evidence that switching between these two experimenters would make a substantial impact in the measurements.

Table 3 *Variance due to each experimental condition and total variance observed.*

Metric	id	session	experimenter	Residual	Total
TSCIS	5.9909	0.3589	0.0026	0.2606	6.6130
OFS	2.1635	0.8646	0.0077	0.2737	3.3095

5 OBSERVATIONS AND LIMITATIONS

No control for the primary outcome

The choice for study design includes two different secondary treatments but there were no control groups for polylaminin without those secondary treatments. This makes it hard to estimate the effect due to polylaminin alone. In this analysis the effect attributed to polylaminin also includes the effect of its conjugation with Chondroitinase ABC, as the reference level for the secondary treatment.

Session is used as a proxy for time

In the supplemental time-varying analysis (section 8.2.2 in the Appendix) a choice was made to use the session indicator instead of the precise amount of time elapsed between observations. This choice takes full advantage of the study design (e.g. in the number of measurements per subject) and simplifies both the data treatment and the interpretation of results. The underlying assumption is that subjects are followed-up at regular intervals and small delays between consecutive sessions may occur. When such scheduling variability is small it should not produce bias in the analysis, so the benefits outweigh the costs.

Recommended reporting guideline

The adoption of the EQUATOR network (http://www.equator-network.org/) reporting guidelines have seen increasing adoption by scientific journals. All clinical trials are

recommended to be reported following the CONSORT guideline (Schulz K F, Altman D G, Moher D., 2010).

6 CONCLUSIONS

When combined with any of the secondary treatments in the study protocol, polylaminin has shown efficacy in the recovery of motor function after spinal cord injury. This data shows an average increase of 1 point in the TSCIS score and 1.6 points in the OFS score.

There is no evidence of a differential efficacy between either Chondroitinase ABC or GDNF as a secondary treatment.

The validation of experimenters show high precision for this design factor, and there is no evidence that switching technicians would bias the results of the analysis. Variance due to experimenters represents at most 0.2% of the total variance in the experiment.

7 REFERENCES

- **SAP-2023-034-TS-v02** – Analytical Plan for Efficacy of polylaminin on the recovery of motor function after spinal cord injury: non-clinical trial
- Sampaio, TLC, (2023). ESTUDO CLÍNICO VETERINÁRIO DO EFEITO DA POLILAMININA ASSOCIADA À CONDROITINASE ABC OU AO FATOR NEUROTRÓFICO DERIVADO DE GLIA EM LESÕES MEDULARES CRÔNICAS EM CÃES
- Schulz K F, Altman D G, Moher D. CONSORT 2010 Statement: updated guidelines for reporting parallel group randomised trials BMJ 2010; 340 :c332 (https://doi.org/10.1136/bmj.c332).

8 APPENDIX

8.1 Exploratory data analysis

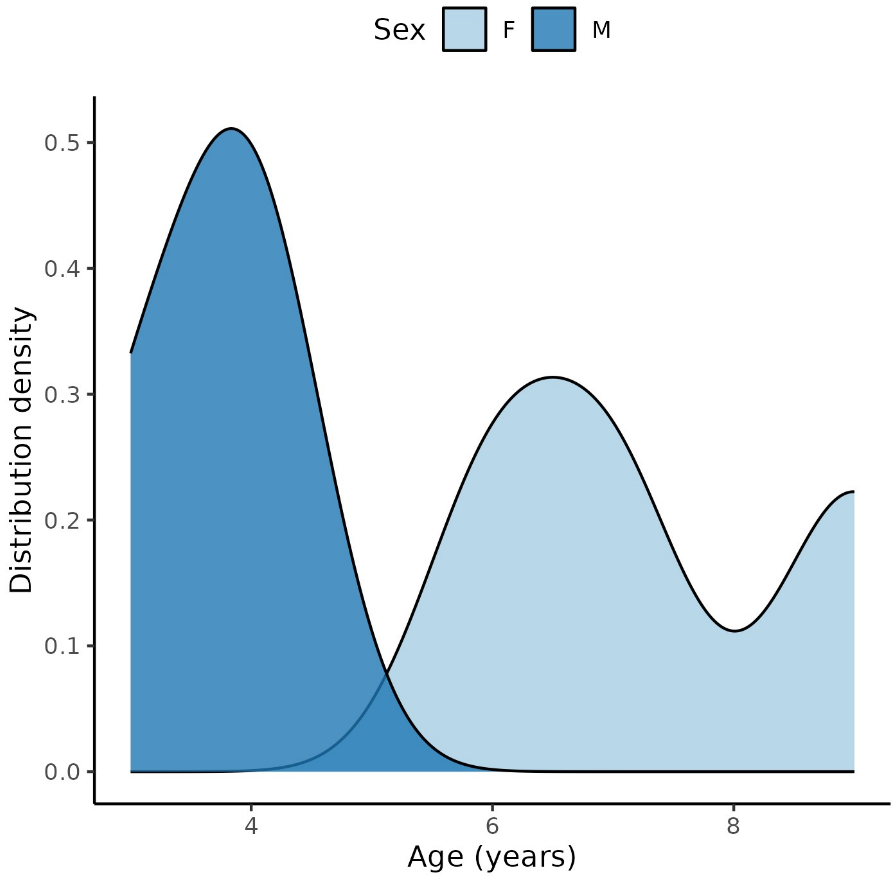

Figure A1 *Distribution of age in the study population.*

8.2 Modeling strategy

8.2.1 Model specification

Several attempts have been made to include as many covariates defined in the SAP as possible. Some variables caused models to fail to converge in most cases, in particular age. This might be due to the extreme variability in that variable (see Figure A1 in section 8.1). Any model specification attempted was required to converge in all four models fitted during this analysis: both average effect and both time-varying effect analyses.

The final model specification could include either multiple lesions or recent lesion covariates but not both; multiple lesions was chosen following the prioritization based on subject-matter expertise provided by the client.

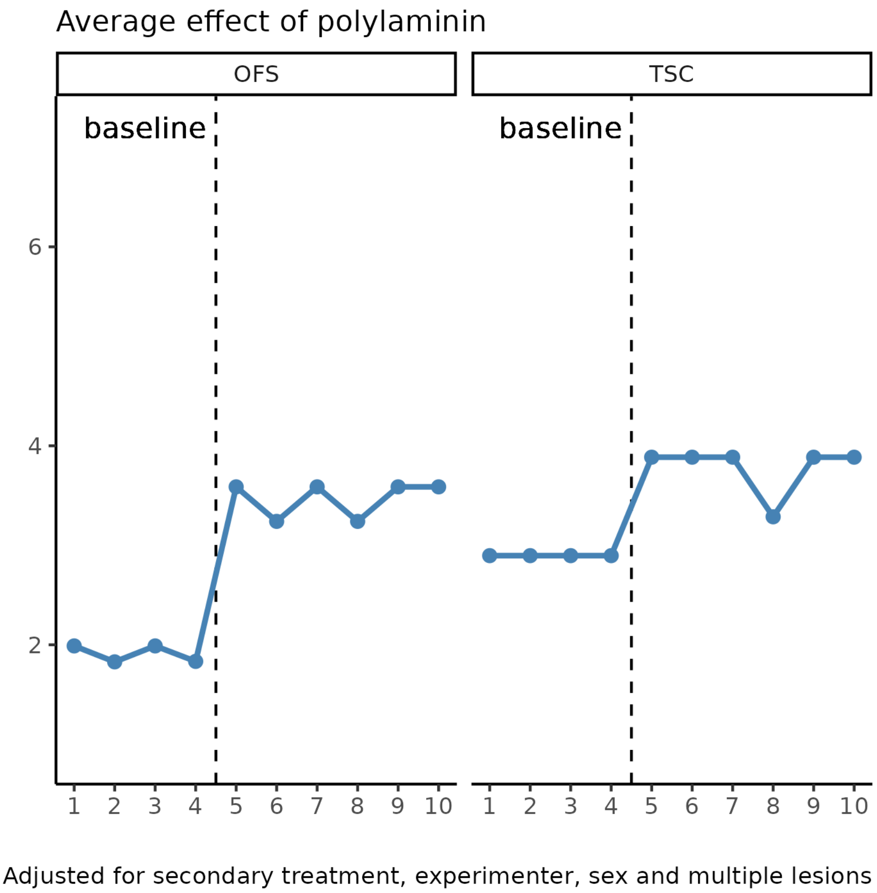

Figure A2 *Effect of the treatment on TSCIS and OFS.*

8.2.2 Time-varying effect

The efficacy measure presented in the body of this report reflects the average effect of the combination treatment investigated during the period defined in the study protocol (Sampaio, 2023). Here we describe a supplemental approach that allows the effect to vary over time, thus measuring the increment in functional scores that can be estimated per follow-up session after the intervention. This was achieved by including an interaction term between the treatment and the session indicator (see Observations and limitations). When those terms are significant, it is customary to disregard the interpretation of the individual terms and describe only the interaction.

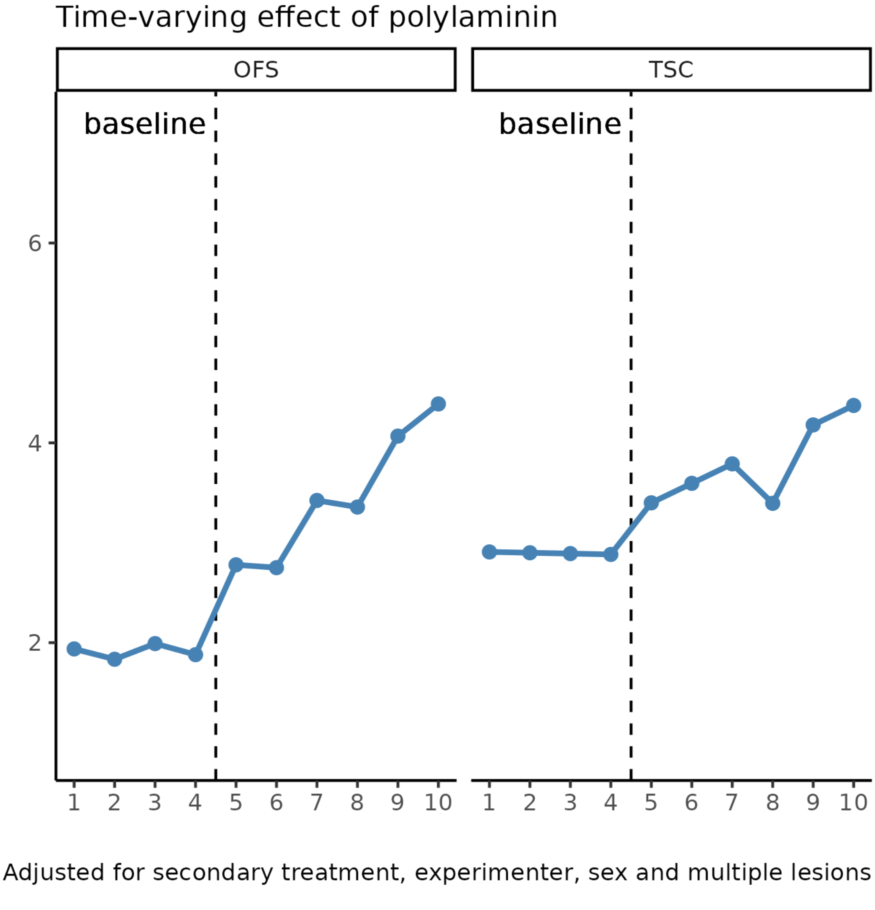

Figure A3 *Time-varying effect of the treatment on TSCIS and OFS.*

The interaction between the treatment and the session was significant for the TSCIS with an increase of 0.20 (95% CI 0.06, 0.34; p=0.005) points per session after the treatment was applied. All other variables kept constant, this means that after 5 follow-up sessions one could expect an increase of one point in the TSCIS scale, after adjusting

for secondary treatment, sex and multiple lesions. The interaction as also significant in the OFS model. After the treatment is applied there is an incremental increase of 0.30 (95% CI 0.15, 0.44; p<0.001) points per session, after adjusting for the other variables. A one point increase in this scale could be expected after four sessions post intervention. Figure A3 shows how the incremental increase impacts the average score for both TSCIS and OFS scales. The intercepts of these models (basal averages described in the body) are contained in Table A2 in the next section.

8.2.3 Full listing of coefficients in all models

Table A1 *Coefficients for both efficacy analyses in this report.*

	TSCIS Average effect			OFS Average effect			TSCIS Time-varying effect			OFS Time-varying effect		
**Characteristic**	**Beta**	**95% CI**[1]	**p-value**	**Beta**	**95% CI**[1]	**p-value**	**Beta**	**95% CI**[1]	**p-value**	**Beta**	**95% CI**[1]	**p-value**
(Intercept)	2.2	-5.7 to 10	0.349	1.5	-3.8 to 6.8	0.349	5.2	-2.8 to 13	0.112	1.5	-3.6 to 6.7	0.344
Treatment	0.99	0.77 to 1.2	**<0.001**	1.6	1.3 to 1.9	**<0.001**	-0.49	-1.1 to 0.11	0.110	-0.74	-1.4 to -0.13	**0.018**
Secondary treatment												
Chondroitinase ABC	—	—		—	—		—	—		—	—	
GDNF	-0.22	-10 to 9.5	0.932	-0.27	-6.8 to 6.3	0.877	3.4	-4.6 to 11	0.210	-0.26	-6.8 to 6.3	0.881
Sex												
F	—	—		—	—		—	—		—	—	
M	2.9	-6.8 to 13	0.327	1.8	-4.8 to 8.4	0.356	-0.75	-8.7 to 7.2	0.723	1.8	-4.8 to 8.3	0.366
Multiple lesions	-1.4	-11 to 8.4	0.600	-0.60	-7.2 to 6.0	0.733	-7.2	-15 to 0.81	0.061	-0.77	-7.3 to 5.8	0.662
Session							-0.01	-0.15 to 0.13	0.907	0.03	-0.12 to 0.17	0.710
Treatment * Session							0.20	0.06 to 0.34	**0.005**	0.30	0.15 to 0.44	**<0.001**

[1]CI = Confidence Interval

8.3 Availability

The client has requested that this analysis be kept confidential until a future date, determined by the client. All documents from this consultation are therefore not published online and only the title and year of the analysis will be included in the consultant's Portfolio. After the agreed date is reached, the documents will be released.

The portfolio is available at:

https://philsf-biostat.github.io/SAR-2023-034-TS/

8.4 Analytical dataset

Table A2 shows the structure of the analytical dataset.

Table A2 *Analytical dataset structure*

id	exposure	ofs	tscis	group	date	session	experimenter	name	sex	age	race	lesion_multiple	lesion_recent
1													
2													
3													
…													
120													

Due to confidentiality the data-set used in this analysis cannot be shared online in the public version of this report.
