# Supplementary material for: A laminin-based therapy for dogs with chronic spinal cord injury: promising results of a longitudinal trial
Source: Front Vet Sci. 2025 Aug 13;12:1592687. doi: 10.3389/fvets.2025.1592687 (PMC12380836; doi:10.3389/fvets.2025.1592687)
Supplement: Supplementary file 3 [file Data_Sheet_3.pdf]

# Figure S1 - Neurological examinations

**Legend:**
Neurological examinations were performed on all dogs immediately before each treatment and at the end of the follow-up period (6 months). Changes over time are color-coded: light pink indicates improvements, and yellow indicates deteriorations.

| **P01** | Before 1st injection | Before 2nd injection | End of follow-up |
|---|---|---|---|
| **Mental status** | | | |
| Consciousness | Alert | Alert | Alert |
| Behavior | Normal | Normal | Normal |
| **Posture and gait** | | | |
| Right hindlimb | Paretic | Paretic | Paretic |
| Left hindlimb | Paretic | Paretic | Paretic |
| **Proprioception** | | | |
| Right hindlimb | Absent | Absent | Absent |
| Left hindlimb | Absent | Absent | Present |
| **Femoral reflex** | | | |
| Right hindlimb | Normal | Normal | Normal |
| Left hindlimb | Normal | Normal | Normal |
| **Patellar reflex** | | | |
| Right hindlimb | Normal | Normal | Normal |
| Left hindlimb | Normal | Normal | Normal |
| **Perineal reflex** | Normal | Normal | Normal |
| **Withdrawal Reflex** | | | |
| Medial digits – RHL | Normal | Normal | Normal |
| Medial digits – LHL | Normal | Normal | Normal |
| Lateral digits – RHL | Normal | Normal | Normal |
| Lateral digits – LHL | Normal | Normal | Normal |
| **Sensory examination** | | | |
| Superficial pain | Absent | Absent | Absent |
| Deep pain | Absent | Absent | Absent |

| **P02** | Before 1st injection | Before 2nd injection | End of follow-up |
|---|---|---|---|
| **Mental status** | | | |
| Consciousness | Alert | Alert | Alert |
| Behavior | Normal | Normal | Normal |
| **Posture and gait** | | | |
| Right hindlimb | Plegic | Plegic | Paretic |
| Left hindlimb | Paretic | Paretic | Paretic |
| **Proprioception** | | | |
| Right hindlimb | Absent | Absent | Absent |
| Left hindlimb | Absent | Present | Present |
| **Femoral reflex** | | | |
| Right hindlimb | Normal | Normal | Normal |
| Left hindlimb | Normal | Normal | Normal |
| **Patellar reflex** | | | |
| Right hindlimb | Normal | Normal | Normal |
| Left hindlimb | Normal | Normal | Normal |
| **Perineal reflex** | Normal | Normal | Normal |
| **Withdrawal Reflex** | | | |
| Medial digits – RHL | Normal | Normal | Normal |
| Medial digits – LHL | Normal | Normal | Normal |
| Lateral digits – RHL | Normal | Normal | Normal |
| Lateral digits – LHL | Normal | Normal | Normal |
| **Sensory examination** | | | |
| Superficial pain | Absent | Absent | Absent |
| Deep pain | Absent | Absent | Absent |

| **P03** | Before 1st injection | Before 2nd injection | End of follow-up |
|---|---|---|---|
| **Mental status** | | | |
| Consciousness | Alert | Alert | Alert |
| Behavior | Normal | Normal | Normal |
| **Posture and gait** | | | |
| Right hindlimb | Plegic | Plegic | Paretic |
| Left hindlimb | Plegic | Plegic | Paretic |
| **Proprioception** | | | |
| Right hindlimb | Absent | Absent | Absent |
| Left hindlimb | Absent | Absent | Absent |
| **Femoral reflex** | | | |
| Right hindlimb | Normal | Normal | Normal |
| Left hindlimb | Normal | Normal | Normal |
| **Patellar reflex** | | | |
| Right hindlimb | Normal | Normal | Decreased |
| Left hindlimb | Normal | Normal | Normal |
| **Perineal reflex** | Normal | Normal | Normal |
| **Withdrawal Reflex** | | | |
| Medial digits – RHL | Normal | Normal | Normal |
| Medial digits – LHL | Normal | Normal | Normal |
| Lateral digits – RHL | Normal | Normal | Normal |
| Lateral digits – LHL | Normal | Normal | Normal |
| **Sensory examination** | | | |
| Superficial pain | Absent | Absent | Present |
| Deep pain | Absent | Absent | Present |

| P04 | Before 1st injection | Before 2nd injection | End of follow-up |
|---|---|---|---|
| **Mental status** | | | |
| Consciousness | Alert | Alert | Alert |
| Behavior | Normal | Normal | Normal |
| **Posture and gait** | | | |
| Right hindlimb | Paretic | Paretic | Paretic |
| Left hindlimb | Paretic | Paretic | Paretic |
| **Proprioception** | | | |
| Right hindlimb | Absent | Absent | Absent |
| Left hindlimb | Absent | Absent | Absent |
| **Femoral reflex** | | | |
| Right hindlimb | Normal | Normal | Normal |
| Left hindlimb | Normal | Normal | Normal |
| **Patellar reflex** | | | |
| Right hindlimb | Decreased | Decreased | Normal |
| Left hindlimb | Normal | Normal | Normal |
| **Perineal reflex** | Decreased | Decreased | Normal |
| **Withdrawal Reflex** | | | |
| Medial digits – RHL | Normal | Normal | Normal |
| Medial digits – LHL | Normal | Normal | Normal |
| Lateral digits – RHL | Normal | Normal | Normal |
| Lateral digits – LHL | Normal | Normal | Normal |
| **Sensory examination** | | | |
| Superficial pain | Absent | Absent | Absent |
| Deep pain | Absent | Absent | Absent |

| P05 | Before 1st injection | Before 2nd injection | End of follow-up |
|---|---|---|---|
| **Mental status** | | | |
| Consciousness | Alert | Alert | Alert |
| Behavior | Normal | Normal | Normal |
| **Posture and gait** | | | |
| Right hindlimb | Paretic | Paretic | Paretic |
| Left hindlimb | Paretic | Paretic | Paretic |
| **Proprioception** | | | |
| Right hindlimb | Absent | Absent | Absent |
| Left hindlimb | Absent | Absent | Present |
| **Femoral reflex** | | | |
| Right hindlimb | Normal | Normal | Normal |
| Left hindlimb | Normal | Normal | Normal |
| **Patellar reflex** | | | |
| Right hindlimb | Normal | Normal | Normal |
| Left hindlimb | Normal | Decreased | Normal |
| **Perineal reflex** | Normal | Normal | Normal |
| **Withdrawal Reflex** | | | |
| Medial digits – RHL | Normal | Normal | Normal |
| Medial digits – LHL | Normal | Normal | Normal |
| Lateral digits – RHL | Normal | Normal | Normal |
| Lateral digits – LHL | Normal | Normal | Normal |
| **Sensory examination** | | | |
| Superficial pain | Absent | Absent | Absent |
| Deep pain | Absent | Absent | Absent |

| **P06** | Before 1st injection | Before 2nd injection | End of follow-up |
|---|---|---|---|
| **Mental status** | | | |
| Consciousness | Alert | Alert | Alert |
| Behavior | Normal | Normal | Normal |
| **Posture and gait** | | | |
| Right hindlimb | Plegic | Plegic | Paretic |
| Left hindlimb | Paretic | Paretic | Paretic |
| **Proprioception** | | | |
| Right hindlimb | Absent | Absent | Absent |
| Left hindlimb | Absent | Absent | Absent |
| **Femoral reflex** | | | |
| Right hindlimb | Normal | Normal | Normal |
| Left hindlimb | Normal | Normal | Normal |
| **Patellar reflex** | | | |
| Right hindlimb | Decreased | Decreased | Decreased |
| Left hindlimb | Decreased | Decreased | Normal |
| **Perineal reflex** | Normal | Normal | Normal |
| **Withdrawal Reflex** | | | |
| Medial digits – RHL | Normal | Normal | Normal |
| Medial digits – LHL | Normal | Normal | Normal |
| Lateral digits – RHL | Normal | Normal | Normal |
| Lateral digits – LHL | Normal | Normal | Normal |
| **Sensory examination** | | | |
| Superficial pain | Absent | Absent | Absent |
| Deep pain | Absent | Absent | Absent |
