# Supplementary material for: A laminin-based therapy for dogs with chronic spinal cord injury: promising results of a longitudinal trial
Source: Front Vet Sci. 2025 Aug 13;12:1592687. doi: 10.3389/fvets.2025.1592687 (PMC12380836; doi:10.3389/fvets.2025.1592687)
Supplement: Supplementary file 4 [file Data_Sheet_4.pdf]

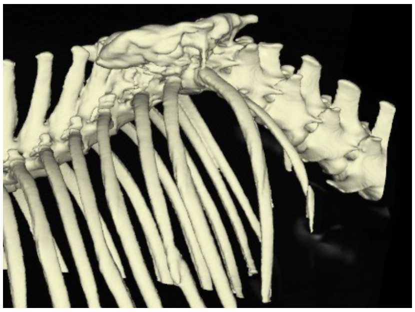

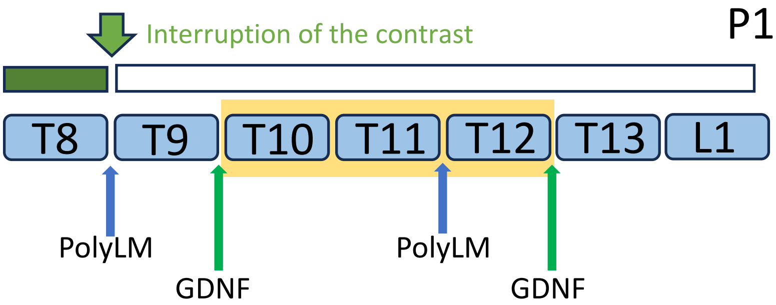

Computed tomography revealed a loss of anatomical alignment of the thoracic spine and disruption of the articular relationship between T10 and T12 (yellow box), along with an interruption in the continuity of the vertebral canal. Myelography showed dorsolateral thinning of the intrathecal contrast column between the vertebral body caudal to T8. As precise location of the parenchymal lesion was not feasible, the polyLM dose was divided: one portion was administered at T8–T9 to address contrast diffusion restriction, and the other at T11–T12 to target the center of the fracture. Given the distance between the polyLM injection sites, the GDNF dose was similarly split and administered into the T9-10 and T12-13 intervertebral spaces, immediately caudal to each polyLM site.

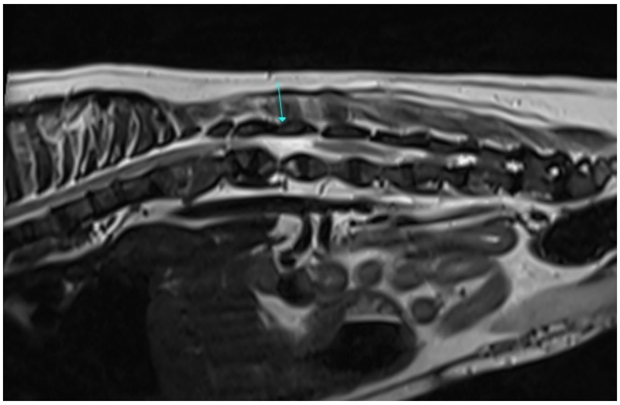

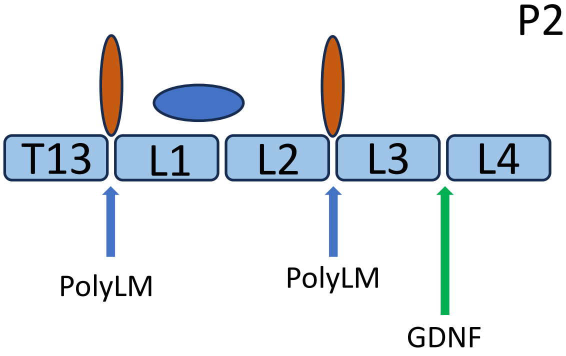

MRI revealed two sites of discal compression at T13–L1 and L2–L3 (brown ovals), along with a 1.8 cm T2-weighted hyperintense intramedullary lesion extending from L1 to the rostral aspect of L2 (blue oval). PolyLM was administered into the T13–L1 and L2–L3 intervertebral spaces to target the parenchymal lesion while avoiding the region suggestive of malacia. GDNF was subsequently delivered into the L3-4 intervertebral space, immediately caudal to the second PolyLM injection.

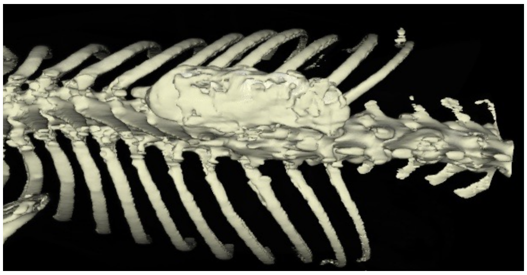

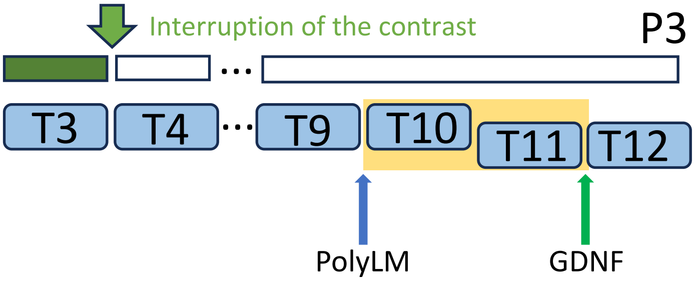

Computed tomography revealed a severe loss of anatomical alignment of the thoracic spine and disruption of the articular relationship between T10 and T11 (yellow box), with marked dorsal displacement of the T11 vertebral body relative to T10. An abrupt interruption in the continuity of the vertebral canal was observed. Myelography demonstrated a complete loss of the intrathecal contrast column caudal to T3. Based on imaging findings, treatment was planned at two sites: T2–3 and T9–10. However, the former site was inaccessible at the time of injection, and the full dose of polyLM was administered into the T9–10 intervertebral space. GDNF was subsequently delivered at the T11–12 intervertebral space to avoid the epicenter of the suspected transection.

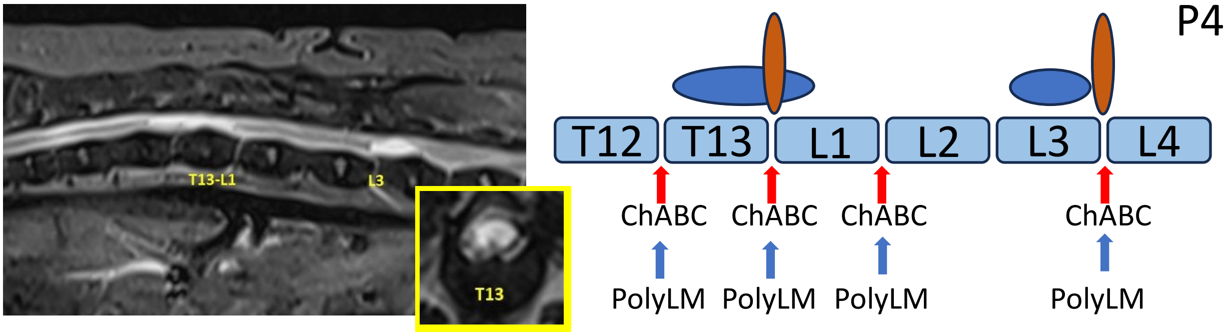

MRI revealed two sites of discal compression at T13–L1 and L3–4 (brown ovals), along with two T2-weighted hyperintense intramedullary lesions: one measuring 2.5 cm, extending from T13 to T13–L1, and another of 1.4 cm, located at L3 (blue ovals). Chondroitinase ABC (ChABC) was administered into the T13-L1 and L3–4 intervertebral discs, corresponding to the compression sites. Due to the marked parenchymal lesion at T13/T13–L1, suggestive of frank malacia, two additional injections were performed to flank the area—at T12–13 and L1–2 —totaling four injections. PolyLM was subsequently delivered into the same intervertebral spaces.

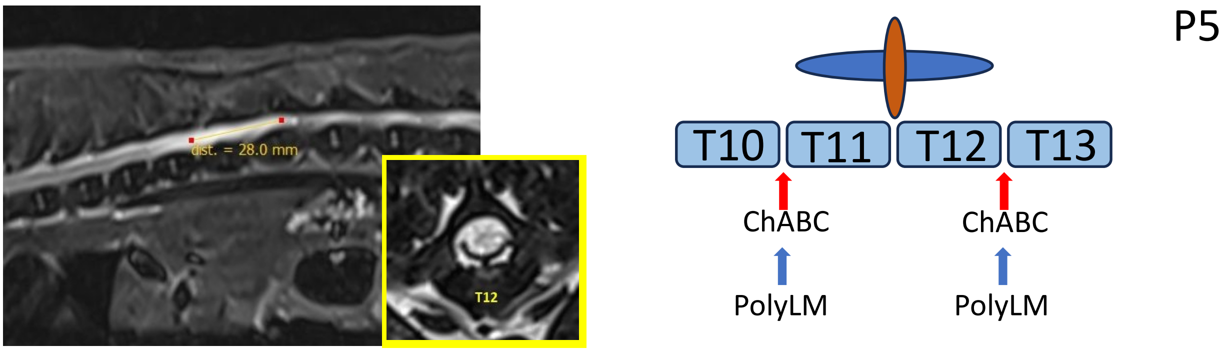

MRI revealed a site of discal compression at T11–12 (brown oval), along with an exuberant T2-weighted hyperintense intramedullary lesion measuring 2.8 cm and extending from T10-11 to T12-13 (blue oval). Chondroitinase ABC (ChABC) was administered into the T10-11 and T12-13 intervertebral discs, flanking the area of extensive malacia. PolyLM was subsequently delivered into the same two intervertebral spaces.

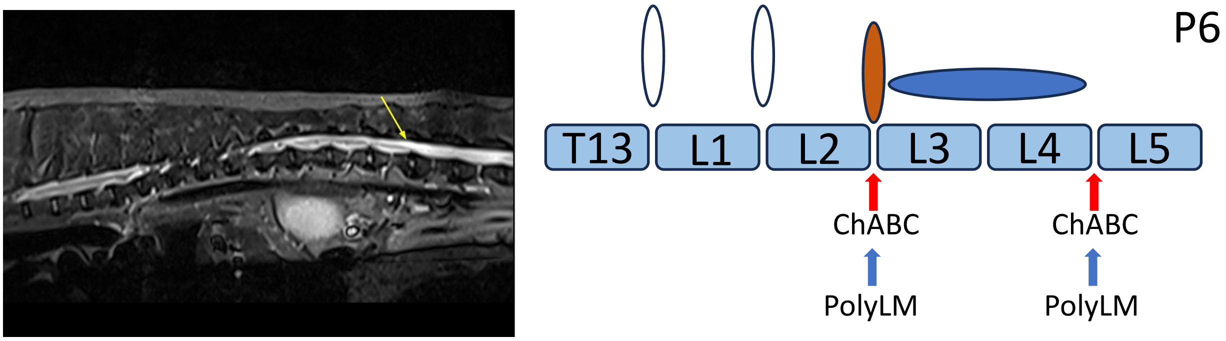

MRI revealed three sites of discal compression at T13–L1, L1–2, and L2–3 (ovals), along with an exuberant T2-weighted hyperintense intramedullary lesion extending from L2–3 to L4 (blue oval). Since the two most rostral compression sites (empty ovals) were not associated with parenchymal lesions, they were not considered responsible for neurological injury. The dose of Chondroitinase (ChABC) was divided into two portions: one administered into the L2–3 intervertebral disc, corresponding to the primary compression site, and the other at L4–5. The rationale for the second injection was twofold: (1) to enhance medication distribution across the extensive parenchymal injury; and (2) to address potential local damage, as suggested by decreased bilateral patellar reflexes observed during the neurological examination (Fig. S1). PolyLM was subsequently delivered into the same two intervertebral spaces.
